# Structure-aware machine learning strategies for antimicrobial peptide discovery

**DOI:** 10.1038/s41598-024-62419-y

**Published:** 2024-05-25

**Authors:** Mariana D. C. Aguilera-Puga, Fabien Plisson

**Affiliations:** Department of Biotechnology and Biochemistry, Center for Research and Advanced Studies of the National Polytechnic Institute (CINVESTAV-IPN), Irapuato Unit, 36824 Irapuato, Guanajuato Mexico

**Keywords:** Explainable machine learning, Peptide design, Oversampling, Structural bias, Protein structure prediction, AlphaFold2, Mechanism of action, Peptides, Protein design, Protein folding, Computational biology and bioinformatics, Drug discovery, Mathematics and computing

## Abstract

Machine learning models are revolutionizing our approaches to discovering and designing bioactive peptides. These models often need protein structure awareness, as they heavily rely on sequential data. The models excel at identifying sequences of a particular biological nature or activity, but they frequently fail to comprehend their intricate mechanism(s) of action. To solve two problems at once, we studied the mechanisms of action and structural landscape of antimicrobial peptides as (i) membrane-disrupting peptides, (ii) membrane-penetrating peptides, and (iii) protein-binding peptides. By analyzing critical features such as dipeptides and physicochemical descriptors, we developed models with high accuracy (86–88%) in predicting these categories. However, our initial models (1.0 and 2.0) exhibited a bias towards α-helical and coiled structures, influencing predictions. To address this structural bias, we implemented subset selection and data reduction strategies. The former gave three structure-specific models for peptides likely to fold into α-helices (models 1.1 and 2.1), coils (1.3 and 2.3), or mixed structures (1.4 and 2.4). The latter depleted over-represented structures, leading to structure-agnostic predictors 1.5 and 2.5. Additionally, our research highlights the sensitivity of important features to different structure classes across models.

## Introduction

Machine learning (ML) models gradually emerge as cost-effective, time-saving, and informed strategies to accelerate the discovery and design of peptides and proteins. They contribute to our understanding of the sequence-structure–function relationships by capitalizing on the abundant protein sequences and more limited structural or functional information. Predictive ML models infer tailored properties or functions like tridimensional structure, protein–protein interactions, target binding affinity, stability, and solubility. Generative ML models create novel biological modalities with the desired properties—i.e., de novo protein design, antibody or enzyme engineering. The combination of predictive and generative models minimizes the need for extensive experiments and resources while increasing our chances of achieving successful outcomes, e.g., drug hits or leads. The main applications of ML models for biofuel production, material design, or drug development include designing peptides with one or more objectives (e.g., cell-penetrating property^1^, antiviral activity^2^, antimicrobial activity^3,4^, anticancer activity^5–8^), protein binders^9–11^, monoclonal antibodies^12,13^, protein families^14–16^, and enzymes^17,18^.

Machine learning models predominantly rely on peptide and protein sequences due to the wealth of information hidden in the linear chains of amino acids. The biological sequences are abundant and easily accessible in public and private repositories, owing to the advancements in genome sequencing, offering a robust basis for training ML algorithms. In addition, sequence-based models are computationally efficient and generalizable, making them an attractive choice for processing sizable datasets to predict the properties of new peptides and proteins. Nevertheless, understanding the sequence-structure–function relationships also requires structural information, a resource existing ML models often lack. This scarcity arises from the elevated costs and resources associated with X-ray crystallography or nuclear magnetic resonance. The last fifteen years of model development for antimicrobial peptides (AMPs)^19–25^ perfectly encapsulate the reliance of machine learning models on peptide and protein sequences.

AMPs, commonly called host-defense peptides (HDPs), are the first lines of defense of many organisms against pathogens acting via direct microbicidal activity or indirect stimulation of the host’s immune responses. They promise to combat global health threats and antimicrobial resistance to conventional antibiotics^26–28^. Most AMPs/HDPs are small amphipathic proteins, generally between 12 and 50 residues, with a net charge between + 2 and + 9 at physiological pH. Despite these common characteristics, the peptides are significantly diverse in sequence and structure. We recently recommended a fast and robust estimate of the structural landscape(s) of medium-large datasets for fold discovery and prior ML modeling. Our predictions identified loose α-helices as the main structure class (65.1%), followed by random coils (17.8%), and β-stranded and mixed structures accounted for the rest of the large AMP dataset^29^. Consequently, current AMP models (predictors and generators) might favor these dominant structure classes.

Antimicrobial peptides have long been thought to kill pathogens uniquely by interacting with their phospholipids, leading to cell death (membranolytic AMPs)^30,31^. Some AMPs/HDPs translocate through cell membranes before reaching intracellular targets (non-membranolytic AMPs)^28^. Their global cationic and amphipathic characteristics are essential to their electrostatic interactions and hydrogen bonding with bacterial and eukaryotic membranes and are inherently linked to their functional promiscuity^32,33^. Current ML models excel at identifying sequences of antimicrobial nature or activity, but they rarely comprehend their intricate mechanism(s) of action. In 2016, Lee and co-workers reported a seminal ML model to understand the relationships between peptide sequences and their interactions with cell membranes^34^. Two years later, Brand and co-workers classified membrane-active peptides by combining the results of differential scanning calorimetry and circular dichroism experiments with unsupervised ML methods^35^. Both original studies uncovered that physicochemical properties (i.e., amphiphilicity, helical propensity) could help predict the peptides’ antimicrobial nature and membrane activity using α-helical peptides. In addition, most biophysical experiments and molecular simulations supporting our understanding of their interactions with lipid membranes predominantly use α-helical probes^36–40^. While these mechanisms apply to most AMPs, the models may not generalize to other structures, and their mechanisms of action remain mainly unresolved.

In the present study, we developed predictive models capable of distinguishing between (i) membrane-active peptides that induce bacterial membrane disruption (MDPs), (ii) those that solely penetrate through the membrane to reach one or more intracellular target(s) (MPPs), and (iii) peptides binding to larger proteins (PBPs). Aware of the possible over-representation of α-helical AMPs in our training datasets, we studied the predictive power of our models against different secondary structures and identified structural bias. We reduced our training sets to tackle imbalanced structural classes and construct models that could predict the three mechanisms of action (i–iii), invariably from their structural diversity.

## Methods

**Figure 1:**
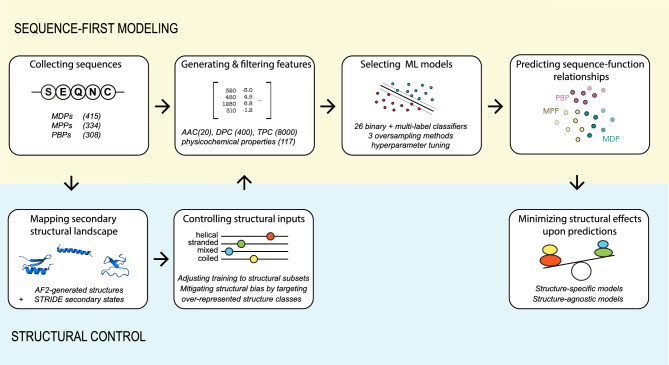
General workflow to predict the functions of peptide sequences with or without considering their structural information. On the top, sequence-first models primarily predict the functions from novel peptides using sequential representation, lacking structural information. On the bottom, we can control any structural bias by mapping the structural landscape of our dataset(s) and constructing structure-specific or structure-agnostic models.

Figure 1 summarizes the general workflow to predict the membrane or protein activity of peptide sequences with or without taking into account their structural information. In sequence-first modeling, we first collected the 1057 peptide sequences from multiple public databases before measuring 8437 features (i.e., compositions in amino acid/dipeptides/tripeptides, global physicochemical properties) for each sequence. Most features were filtered out, and only the most important properties were used to construct machine learning predictors. We evaluated the performances of 12 algorithms for binary classification and 9 algorithms for ternary classification (to distinguish between MDPs, MPPs, and PBPs). After optimizing our models with tenfold cross-validation, oversampling methods, and hyperparameter tuning, we tested them with an external validation set. We explored the structural landscapes of our datasets to discover a strong bias towards a specific structural class; α-helices. We revised our training sets to either limit our predictive model to that structural class or develop a more generalized model by generating native-like sequences from minor structural classes.

### Datasets

#### Model datasets

We searched primary peptide sequences from four publicly available databases; DBAASP v3 (Database of Antimicrobial Activity and Structure of Peptide, https://dbaasp.org/)^41^, APD3 (Antimicrobial Peptide Database)^42^, PDBe (Protein Data Bank in Europe)^43^ and CPPsite 2.0 (Cell Peptide Penetrating site)^44^ using the specified keywords. All peptides were in their monomeric forms, without unusual amino acid modifications, targeted “gram-positive and gram-negative bacteria” and were expected to fold into “α-helix, β-sheet, random coil, and mixtures thereof”. We categorized and assigned all sequences into subsets (labels) based on their mechanisms of action: membranolytic/membrane-disrupting peptides (MDPs) target only “lipid bilayer(s)” of the aforementioned binding targets, while non-membranolytic/membrane-penetrating peptides (MPPs) bind to “DNA, RNA, cytoplasmic protein”. MPPs also include sequences reported as cell-penetrating peptides (CPPs) targeting microbial membranes. MDPs and MPPs form membrane-active peptides (MAPs). The last subset comprises protein-binding peptides (PBPs) that bind to larger eukaryotic protein receptors. For MDPs and MPPs, we utilized the DBAASP v3 and APD3 databases, selecting sequences with activities against both “anti-Gram + and anti-Gram– bacteria”, ensuring that peptides were under 10 kDa and using keywords like “cell-penetrating peptide”, “DNA”, and “RNA” for binding targets. The CPPsite 2.0 database, which has a unique mechanism, was employed for certain MPPs, applying filters like sub-cellular localization type “nucleus” or “cytoplasm”. We only considered those without N and C termini modifications, L-type chirality, and no chemical modifications. Finally, using the PDBe interface, we searched for peptides with terms such as “neuropeptides” and “immunomodulators”, focusing on those that bind to proteins. This comprehensive search also included a literature review to verify activities and mechanisms, thus enhancing our collection of sequences. In total, we gathered 1057 peptides, including 415 MDPs, 334 MPPs (749 MAPs), and 308 PBPs.

#### External validation dataset

We collected 262 peptide sequences that belong to one of the aforementioned classes (72 MDPs, 57 MPPs, or 133 PBPs), but they were not present in the model datasets.

### Features

#### Amino acid composition (AAC)

The Amino Acid Composition (AAC) is the fraction of each amino acid type within a peptide sequence of length N. The AAC values were measured using *iFeature* web server^45^.

#### Dipeptide and Tripeptide composition (AAs and AAAs)

We measured a total of 400 dipeptide features and 8000 tripeptide features from peptide sequences using *iFeature* web server^45^.

#### Physicochemical properties (PCPs)

We measured a total of 118 PCPs from primary peptide sequences; 76 properties with the R package *Peptide* (v.2.4.1)^46^, 33 with the Python package *modlAMP* (v.3.7.3)^47^, and 8 with DBAASP v3^41^. For the definitions of all PCPs, see Lists S1-S3.

### Data pre-processing

All peptide sequences with duplicated information and/or missing values were removed. All model and validation datasets were normalized as X values. The external validation and α-helical datasets were normalized as X_validation_ values relative to the model dataset in use for predictions—see Supporting Information Eqs. (S1)–(S2).

### Feature elimination

#### Multicollinearity

We reduced the number of variables/features (e.g., physicochemical properties) associated with each class to keep only the most informative and non-redundant ones using multicollinearity^48^. Multicollinearity (MC) excludes all highly correlated features to keep only non-redundant properties. In our study, we eliminated redundant properties using a Pearson correlation coefficient cut-off of 0.90.

#### Statistical tests

For statistical analysis of the datasets, we used the methodology described by our group in 2020^49^. To assess and compare the statistical distribution between model datasets through their physicochemical features. We measured the normality of dataset distributions for each class of binary classification models using Shapiro–Wilk and Lilliefors tests, according to the size of the samples, before evaluating which dataset(s) had the same distribution in both groups. We determined the variance with either the F-test for a normally distributed dataset (ND) or the Fligner-Killen test for an abnormally distributed dataset (AD). We compared the means of physicochemical properties between the two classes by applying the three respective statistical tests; (1) Welch’s t-test to NDs with different variances, (2) Wilcoxon test (also known as Wilcoxon rank-sum) to ADs with the same variance and (3) Kolmogorov–Smirnov test to ADs with different variances, using a significance level α of 0.05. We controlled the false discovery rate with the Benjamini and Hochberg method using the same value α. All tests were performed using R (v.3.6.3)^50^ and RStudio^51^. The statistical pipeline is visible in Figs. S3 and S4.

### Building classifiers

#### Machine learning algorithms

We evaluated 12 binary classification algorithms and 9 ternary (multi-label) classification algorithms to discriminate between membrane-disrupting peptides and membrane-penetrating peptides and/or protein-binding peptides. The binary classification algorithms include RFC: Random Forest Classifier^52^, GBC: Gradient Boosting Classifier^53^, ABC: Adaptive Boosting Classifier^54^, LDA: Linear Discriminant Analysis^55^, LR: Logistic Regression^56^, DT: Decision Tree^57^, K-NN: K-Nearest Neighbors^58^, GNB: Gaussian Naïve Bayes^59^, and SVC: Support Vector Classifier (with the 4 kernels: linear, radial basis function, polynomial, sigmoid)^60^. The ternary classifiers include ETC: Extra-Trees Classifier^61^, MNB: multinomial Naïve Bayes^62^, RNC: Radius Neighbors Classifier^63^ in addition to the aforementioned RFC, GBC, LDA, DT, KNN, and GNB. All the models were computed using Python package *scikit-learn* 0.23.1^64^.

#### Performance metrics

We used different metrics to compare the performance of our classification models: accuracy (Acc.), precision (Prec.) or positive predictive value (PPV), recall or true positive rate (TPR), F1 score, Matthews correlation coefficient (MCC), Cohen's Kappa statistic (CK or κ), and the area under the curve Receiver Operating Characteristic (AUC-ROC) value—see Supporting Information Eqs. (S3–S8).

#### Class membership and class probabilities

Each peptide sequence was output a class membership and a class probability P. For binary classifiers, the class membership is either class 0: MPP or 1: MDP), and the class probability P to belong to that same class varies between 0.00 and 1.00 (e.g., P_MDP_ = 0.78). For multiclass classifiers with 3 classes, class membership is either class 0: MPP or 1: MDP or 2: PBP), and the class probability P to belong to that same class varies between 0.00 and 1.00. For each sequence, the sum of class probabilities in each model is equal to 1 (i.e., binary: P_MPP_ + P_MDP_, ternary: P_MPP_ + P_MDP_ + P_PBP_).

#### Tenfold cross-validation

All model datasets were split into two subsets; 1 training dataset (80%) used for model building and 1 smaller testing dataset (20%) used for internal validation. We evaluated the performances of our classification models using a tenfold (k = 10) cross-validation with each subset where sequences are randomly divided into 10 subsets (folds); 9 sets train the models, and the remaining set is for evaluation.

#### Oversampling methods

The three subsets (MDPs, MPPs, and PBPs) form imbalanced classes in their respective model datasets. We implemented three oversampling methods SMOTE (Synthetic Minority Oversampling Technique)^65^, ROSE (Random Over-Sampling Examples)^66^ and ADASYN (Adaptive Synthetic Sampling)^67^ to correct the imbalance by duplicating sequences from the minority class(es).

### Secondary structure prediction

We predicted the tridimensional structures of all peptide sequences (model and external validation datasets) using ColabFold^68^, a handy interface implementing the Alpha Fold 2 (AF2)^69^ technology within the Google Colab environment—version 1.4. The batch mode allows the prediction of multiple peptide sequences in a single session. We requested a single model per sequence through 3 recycles. We kept the AF2 predictions with the highest pLDDT (local distance-dependent transition) and pTM (predicted template modeling) scores. Most models presented pLDDT scores above 80. We submitted all resulting models (.pdb files) to STRIDE^70^ to assign the secondary structure states—% helix (H), % sheet (E), and % coil (C).

### Structural landscape representation and segmentation

We displayed the collective secondary structures of all peptide sequences as single points on a ternary representation—the structural landscape, as previously described^29^. The three axes represent the three secondary structure states—% helix (H), % sheet (E), and % coil (C). We used *ggtern* and *ggplot* libraries^71^ with R (v. 4.3.1) in R Studio environment^51^. In addition to the plots, we quantified the distribution of the secondary structures using a kernel density estimate. To ease readership and quantification, we subdivide the structural landscape into arbitrary regions based on their percentage composition of secondary structure states. In the present study, we created four regions (I) predominantly α-helical peptides, (II) predominantly stranded (β-sheet) peptides, (III) predominantly coiled peptides, and (IV) mixed structures.

## Results

### Identifying key features between membrane-active and protein-binding peptides

Machine learning (ML) models primarily rely on several factors, including the quality of their input data and independent features. First, we carefully curated 1,057 peptide sequences from four public databases (DBAASP v3^41^, APD3^42^, PDBe^43^, CPPsite 2.0^44^) before labeling each sequence with one of the following classes: membrane-disrupting peptides (MDPs, n = 415), membrane-penetrating peptides (MPPs, n = 334), and protein-binding peptides (PBPs, n = 308). The first two classes formed the membrane-active peptides (MAPs, N = 749). Before building models, it was essential to identify and select critical properties that may distinguish between membrane-active peptides and protein-binding peptides. Therefore, we measured 8,537 sequence-derived properties: 20 amino acid fractions, 400 dipeptides, 8000 tripeptides, and 117 physicochemical properties from different sources for all peptides.

**Figure 2:**
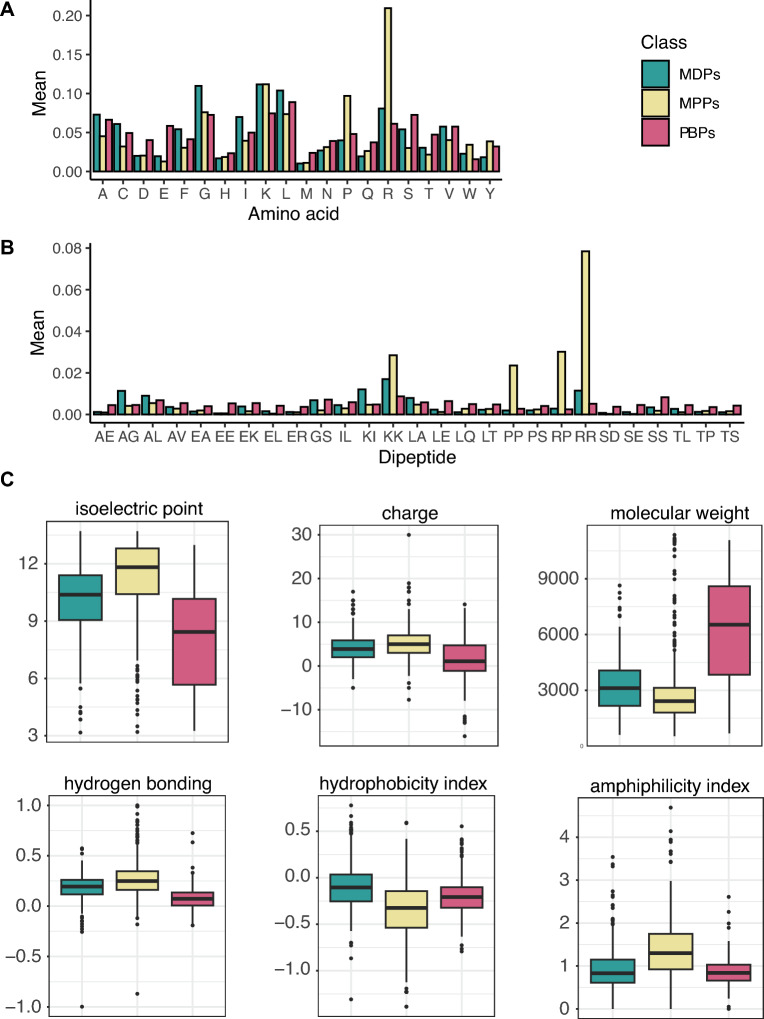
Comparative analyses of amino acid composition (**A**), dipeptide composition (**B**), and global physicochemical properties (**C**) between membrane-disrupting peptides (MDPs, teal green), membrane-penetrating peptides (MPPs, peach yellow), and protein-binding peptides (PBPs, raspberry red).

We first compared the differences in amino acid composition (AAC) between MDPs, MPPs, and PBPs. In Fig. 2A, membrane-penetrating peptides (MPPs, in peach yellow) showed higher contents in proline (0.12) and arginine (0.22) than membrane-disrupting peptides (MDPs, in teal green), which are characteristic of peptides with non-lytic mechanisms, such as cell-penetrating peptides. Both MPPs and MDPs displayed elevated levels of lysine, reminiscent of MAPs. Membrane-disrupting and protein-binding peptides (PBPs, raspberry red) contained many small and aliphatic residues, i.e., alanine, glycine, leucine, and valine. PBPs are enriched in polar and negatively charged residues, i.e., serine, threonine, glutamic and aspartic acids. These polar and charged amino acids indicate the hydrogen bonds and ionic interactions the peptides form with larger protein domains. In contrast, lysine and arginine in MDPs and MPPs suggested that the peptides primarily interacted with negatively charged lipid heads across cell membranes. We repeated the exercise by comparing the composition of dipeptides (DPC) and tripeptides (TPC) across MDPs, MPPs, and PBPs. Due to their enormous proportions of dipeptides (400) and tripeptides (8000), we only kept the crucial differences between membrane-active and protein-binding peptides, as illustrated in Fig. 2B. The presence of positively charged dipeptides “KK”, “RP”, and “RR” in MPPs (in peach yellow) further supported their roles in interacting with lipid membranes. Proline-rich dipeptides “RP” and “PP” are also distinctive features of these membrane-active peptides. Membrane-disrupting peptides (in teal green) are characterized by high levels of alanine-rich and leucine/isoleucine-rich dipeptides—“AG”, “AL”, “IL”, “KI”, “LA”. Finally, protein-binding peptides (in raspberry red) presented the polar and negatively charged dipeptides—“EA”, “EE”, “EK”, “EL”, “ER”, “GS”, “LE”, “LQ”, “LT”, “PS”, “SD”, “SE”, “SS”, “TL”, “TP” and “TS”, crucial to their interactions with larger protein domains. The same analysis was conducted across the 8000 tripeptides; none appeared relevant between the three peptide classes.

Considering the differences in amino acid and dipeptide compositions between the three classes, we measured 117 global physicochemical properties for the 1,057 sequences, 76 properties with the R package *Peptide* (v.2.4.1)^46^, 33 with the Python package *modlAMP* (v.3.7.3)^47^, and 8 with DBAASP v3^41^. The properties are defined in Lists S1-S3. Many of these features encoded for the same peptide property (e.g., hydrophobicity); therefore, we performed correlation analyses using the Pearson correlation coefficient using the datasets from 2 or 3 classes (Figs. S1, S2). Several properties like hydrophobicity scales presented strong positive (in red) or negative correlation (in blue) with one another, suggesting highly redundant information. We eliminated all properties showing a correlation coefficient > 0.90, reducing the number of global physicochemical properties to 53 for the two classes and 57 for the three classes. Moreover, we eliminated irrelevant properties by keeping those that differ between membrane-active and protein-binding peptides. We conducted pairwise statistical analyses across two datasets (MDPs and MPPs, MAPs and PBPs)—see Fig. S3. The first pair should indicate properties linked to membrane activity, whereas the second pair should lead to properties for membrane or protein recognition.

We identified 49 significant properties between MDPs and MPPs, and 56 significant properties between MAPs and PBPs, summarized in Table S1. Two of the 49 properties (i.e., the hydrophobic moment and the hydrophobicity index on the Wilson scale) are exclusively associated with membrane activity, distinguishing between MDPs and MPPs—see properties 1 and 2 in Table S1. The other 47 properties played a role in discerning between membrane-active and protein-binding peptides. Nine properties (i.e., the prevalence of basic and aromatic residues, differences in charged residues, the linear moment, the isoelectric point (pI), and hydrophobicity indices from different scales) participated in differentiating between MAPs and PBPs—see properties 3–11 in Table S1. We illustrated some of these differences in Figs. 2 and S4. Higher isoelectric points, electrophilicity indices, and net charges of membrane-active peptides evoked their abundant basic residues and dipeptides. Membrane-disrupting peptides (in teal green) are generally more hydrophobic than membrane-penetrating peptides (in peach yellow); they displayed higher means for most indices or scales linked to hydrophobicity. These observations are somehow associated with the nature of the peptides, which reside longer within cell membranes before aggregating to form pores. Finally, protein-binding peptides (in raspberry red) are heavier and contain more acidic, aliphatic, and aromatic residues than MPPs, explaining their lower pI (Fig. 2). They also showed higher means for penetration depth and in vitro aggregation propensity (Fig. S4).

### Building baseline binary and ternary classification models

Multiple global physicochemical properties or residues and dipeptides could differentiate between membrane-active and protein-binding peptides, endorsing the development of machine-learning predictors for membrane disruption, membrane penetration, and protein recognition. We selected 12 binary classification algorithms to predict the membrane activity of peptide sequences between MDPs and MPPs and 9 ternary classification algorithms to distinguish between the MAP classes and PBPs. We also corrected the imbalance between the two or three classes (MDPs, MPPs, and PBPs) by duplicating or generating synthetic sequences from the minority class(es) using the three oversampling methods ROSE (Random Over-Sampling Examples)^66^, SMOTE (Synthetic Minority Oversampling Technique)^65^, and ADASYN (Adaptive Synthetic Sampling)^67^. Our best initial results are summarized in Table 1. Additional performances of all classifiers under the three oversampling methods are listed in Supporting Information Tables S2–S4 for binary classification and Tables S5–S7 for ternary classification.

**Table 1:** Performances (mean accuracies in percentage) of 12 binary and 9 ternary classification algorithms to distinguish between membrane-disrupting peptides (MDPs, 415) and membrane-penetrating peptides (MPPs, 334), and protein-binding peptides (PBPs, 308) using either 49 or 56 sequence-based physicochemical descriptors under the different oversampling methods.

Binary classifiers	Oversampling	Train	Test	Ternary classifiers	Oversampling	Train	Test
**RFC (model 1.0)**	**ROSE**	**88.0**	**83.3**	**RFC (model 2.0)**	**ROSE**	**86.7**	**83.5**
GBC	ROSE	86.7	85.3	ETC	ROSE	78.7	70.3
ABC	ROSE	83.4	78.6	GBC	ROSE	85.5	79.7
LDA	ROSE	77.5	76.0	DT	ROSE	77.9	68.4
LR	ROSE	78.7	82.0	KNN	ROSE	78.3	76.8
DT	ROSE	82.1	77.3	RNC	ROSE	33.6	33.5
KNN	ROSE	84.4	79.3	GNB	ROSE	71.2	74.0
GNB	ROSE	78.2	78.0	MNB	ROSE	67.8	75.4
SVC (kernel: RBF)	ROSE	82.7	83.3	LDA	ROSE	74.0	75.0
SVC (linear)	ROSE	78.9	80.0	–	–	–	–
SVC (polynomial)	ROSE	85.2	83.3	–	–	–	–
SVC (sigmoid)	ROSE	54.1	60.0	–	–	–	–

All performance metrics (accuracy, precision, Matthews correlation coefficient, Cohen’s kappa statistic and receiver operating characteristic area under curve) are available in Supporting Information S1. The best classifiers and their respective performances are depicted in bold.

*RF* random forest, *GBC* gradient boosting classifier, *ABC* adaboost classifier, *LDA* linear discriminant analysis, *LR* logistic regression, *DT* decision tree, *KNN* K-nearest neighbor, *GNB* Gaussian Naïve Bayes, *SVC* support vector machine, *RBF* radial basis function, ETC extra tree classifier, *RNC* radius neighbors classifier, *MNB* multinomial Naïve Bayes.

Overall, binary and ternary models based on the Random Forest Classification (RFC) algorithm outperformed all other classification models, irrespective of the oversampling technique employed. Table 1 showed that combining RFC with the ROSE method led to the highest prediction accuracies to distinguish between the two classes (88.0% and 83.3%) and three classes (86.7% and 83.5%) for training and testing datasets, respectively. Other classifiers based on tree-based algorithms, including Gradient Boosting (GBC), Adaptive Boosting (ABC), Extra Tree (ETC), and Decision Tree (DT) followed suit. GBC-based predictors achieved the second-highest performances with cross-validated training accuracies of 86.7% and 85.5% for binary and ternary classification. DT-based models showed the lowest performances between classifiers using tree-based algorithms. ABC-based binary and ETC-based ternary classifiers displayed intermediate accuracies. With other algorithms, models based on K-nearest neighbor (KNN) and Support Vector Classification (SVC) with polynomial kernel demonstrated relatively good performances in binary classification tasks, with training accuracies ranging between 82.7 and 85.2%. These observations support the recent statement that tree-based models RFC and GBC performed very well in classifying tabular data^72^. Previous studies have previously demonstrated that tree-based models outperformed other algorithms in classification or regression tasks using *modlAMP* descriptors^49,73–75^ or other features^76^.

Oversampling methods ROSE, SMOTE, and ADASYN are widely used to address the class imbalance in classification tasks. ROSE selects and duplicates sequences from the minority class (e.g., MPPs in binary classifiers)^66^. In SMOTE, the method generates synthetic sequences interpolated from the minority class^65^. ADASYN is an extension of the SMOTE oversampling method that adapts the number of synthetic sequences generated from the minority class based on varying degrees of imbalance^67^. The performances of our classifiers appeared to consistently follow the same order; RFC-based models outperformed all other classifiers, followed by GBC-based and KNN-based models, regardless of the classification task and oversampling method. Binary models (Tables S2–S4) and ternary models (Tables S5–S7) using the oversampling method ROSE yielded the best results through tenfold cross-validation. The performances of classifiers using SMOTE and ADASYN remained relatively close. For example, our best binary classifier using the RFC algorithm showed cross-validated training accuracies of 88.0% with ROSE and 86.5% with both SMOTE and ADASYN methods (Tables S2–S4). ROSE randomly selects minority class(es) sequences and generates new synthetic samples within the space. This randomness helps mitigate biases in the target minority class(es)^66^ that may arise with the other two oversampling methods. This feature was sufficient to achieve a good balance. In general, our classifiers showed a good fit, with training accuracy slightly higher than testing accuracy. In some cases, the models using Logistic Regression (LR) and SVC with radial basis function, linear or sigmoid kernels presented training accuracies lower than testing accuracies under any oversampling method (Table 1, Tables S2–S7). Therefore, we considered binary and ternary classifiers using the RFC algorithm, our models of choice, to further advance our study.

Feature importances extracted from tree-based algorithms are essential for model interpretability and improvement in predictive science. In our hands, they provided valuable insights into the underlying relationships between peptide descriptors and their mechanisms of action (i.e., classes). We can visualize the most relevant descriptors for our binary and ternary RFC models in Fig. 5 and Tables S8, S9. In Fig. 5A (model 1.0), elements of hydrophobicity (*hydrophobic ratio*, *H. index*), size (*molecular weight*), amphiphilicity (*aliphatic amino acids*, *% tiny residues*, *flexibility*, *ABHPRK*), and charge (*charge density*, *electrophilicity*) were among the key features separating membrane-disrupting peptides (MDPs) from membrane-penetrating peptides (MPPs). This is consistent with recent studies highlighting the role played by amphiphilicity in the membrane activity of α-helical AMPs^34,35^. In Fig. 5B (model 2.0), molecular weight and charge density were the most critical descriptors to differentiate the two classes mentioned above and PBPs. Differences in isoelectric points and hydrogen bonding played minor roles between the two or three classes. These observations are reminiscent of the differences in Figs. 2C and S4.

### Auditing the datasets for structural bias

**Figure 3:**
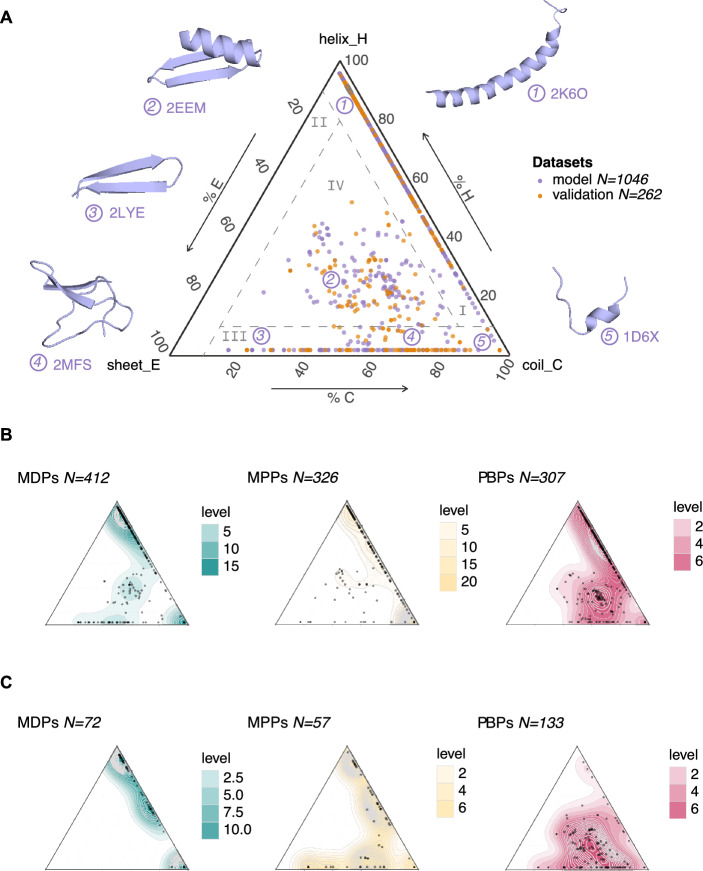
The structural landscapes of our model and external validation datasets. (**A**) Ternary plot illustrating the structural compositions of the model (purple) and external validation (orange) datasets using AlphaFold2 + STRIDE secondary structure predictions for the 3 states helix (H), sheet (E), and coil (C), expressed in percentages. The following five examples, colored in purple, serve as structural markers: (1) human LL-37 (PDB ID: 2K6O), (2) synthetic mytilin (2EEM), (3) θ-defensin BTD-2 (2LYE), (4) cactus-derived Ep-AMP1 (2MFS), and (5) tritrpticin (1D6X). The plot has been partitioned into 4 structural regions (I–IV). (**B**) and (**C**) Ternary plots showing the structural compositions of each subset—MDPs (teal green), MPPs (peach yellow), and PBPs (raspberry red)—in the model and external validation datasets, respectively.

**Figure 4:**
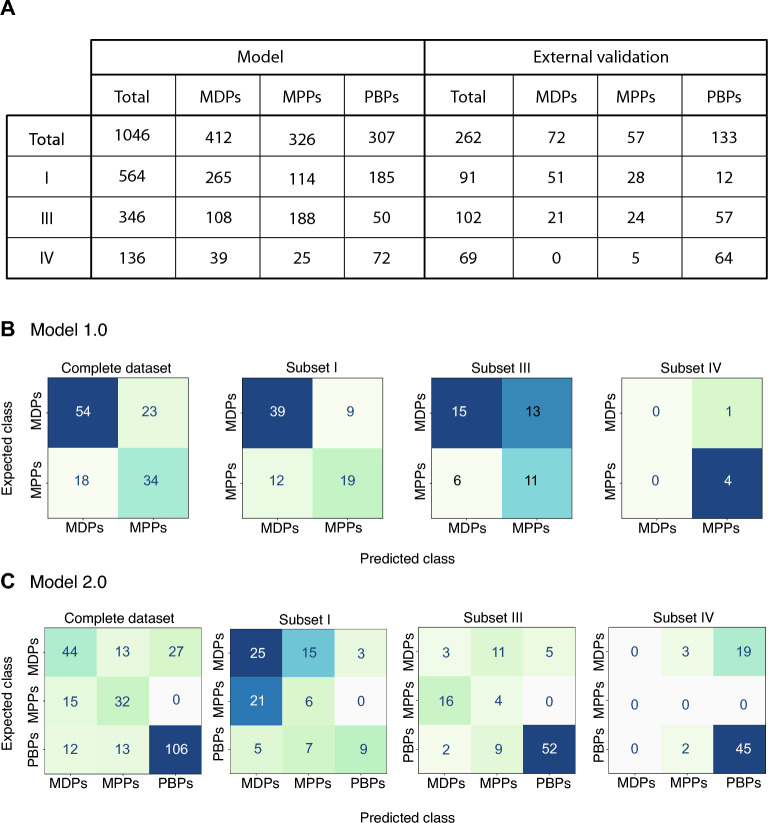
(**A**) Repartitions of the predicted AF2 + STRIDE structures within structural regions (I-IV) for the model and external validation datasets across the three classes; membrane-disrupting peptides (MDPs), membrane-penetrating peptides (MPPs), and protein-binding peptides (PBPs). (**B**,**C**) Confusion matrices of the external validation as a whole and its subsets (I-IV), according to binary and ternary classifiers (models 1.0 and 2.0), respectively.

Aware of the possible over-representation of α-helical peptides in our models, we evaluated the structural diversity of the peptide datasets. We recently developed a fast and reliable approach to estimate the structural landscape of any sizable peptide dataset, using protein structure predictors, PEP2D and AlphaFold2 (AF2)^29^. Many of the sequences in our datasets contained 50 or more residues, guiding our preferences for AF2. Thus, we predicted the tridimensional structures of all peptide sequences using the ColabFold environment with AF2 in batch mode through 3 recycles^68^. A few structures were incorrectly predicted and were ignored, leading to a final model dataset of 412 MDPs, 326 MPPs, and 307 PBPs. Our resulting predicted structures were submitted to STRIDE^70^ to assign the secondary structure states—% helix (H), % sheet (E), and % coil (C). We displayed the global and class-specific structural compositions of our model and external validation datasets in Fig. 3. To ease readership and countability, we divided the ternary plots into 4 structural regions, namely (I) predominantly helical peptides, (II) predominantly stranded (β-sheet) peptides, (III) predominantly coiled peptides, and (IV) mixed structures. The sizes of peptide subsets across structural regions and classes are summarized in a table (Fig. 4A).

In Fig. 3A, we observed the structural landscapes of the model dataset with 1046 peptides (purple) and the external validation dataset, including 262 AF2 + STRIDE predictions (orange). Both datasets are distributed across three of the four structural regions (I, III, and IV), none displaying predicted stranded (β-sheet) peptides (II). For the model dataset, most predictions assumed helical structures with varying coiled levels in the region (I), i.e., 564 peptides (53.9%), followed by 346 coiled peptides (III: 33.1%) and 136 mixed structures (IV: 13.0%). The external validation set presented nearly the exact proportions of α-helices (I: 91, 34.7%) and coils (III: 102, 38.9%), and mixed structures accounted for the rest (IV: 69, 26.4%). In Fig. 3B,C, we reported the respective structural compositions of model and external validation datasets per class—MDPs (teal green), MPPs (peach yellow), and PBPs (raspberry red). Ternary plots in Fig. 3B confirmed that most sequences in the model dataset would fold into α-helical peptides (564, 53.9%) and coils (346, 33.1%). A minority of sequences adopted mixed structures (136, 13.0%). In Fig. 4A, structural region (I) presented α-helices among its three classes with 265 MDPs (64.3%), 114 MPPs (34.9%) and 185 PBPs (60.3%). A quarter of MDP sequences (108, 26.2%), more than half of MPPs (188, 57.7%), and some PBPs (50, 16.3%) would be predicted as coiled structures (III). Finally, the three classes also included sequences that would fold into mixed structures: 39 MDPs (9.5%), 25 MPPs (7.7%), and 72 PBPs (23.4%).

These observations suggested that our classification models (Table 1) might predict the mechanisms of action of peptide sequences that would likely fold into α-helices or coils with greater accuracy. We tested this hypothesis by splitting the external validation dataset according to its secondary structures (Fig. 3C) and comparing the performances of RFC models against the whole dataset and its structural subsets (I, III, and IV). The external validation dataset also contained sequences that would adopt folds located in regions (I) and (III), counting 91 α-helices (34.7%) and 102 coiled structures (38.9%) among its three classes (Fig. 4A). In contrast, the dataset was devoid of mixed structures with MDP activity. Most mixed structures were within the class PBPs (64, 92.7%) and few MPPs (5). The results showed the confusion matrices resulting from the binary and ternary classification of 129 peptides in Fig. 4B,C. We defined the misclassification rate as the fraction of incorrectly labeled sequences. For example, in Fig. 4B, our binary classifier (model 1.0) correctly classified 88 peptides as either MDPs (54) or MPPs (34), leading to a misclassification rate of 0.318 (41 out of 129) on the complete dataset. In the same figure, the misclassification rate among α-helical peptides (subset I) was lower, with a value of 0.265, whereas the fraction of misclassified coiled sequences (subset III) reached 0.422. Four-fifths of mixed structures were correctly classified in subset IV. In Fig. 4C, our ternary classifier (model 2.0), roughly a third (0.305) of all sequences were misclassified. The misclassification rate peaked at 0.560 in subset I, predominantly from α-helical MPPs (21 out of 51 misclassified peptides). Among coiled and mixed structures (subsets III and IV), most PBPs (52 out of 57, 45 out of 64) were correctly labeled; the misclassified rates of 0.421 and 0.347 resulted from misclassified membrane-active peptides. The structural imbalance between model subsets partly explained these values; most MDPs were α-helices (265), and half of the PBPs (72) folded into mixed structures, as depicted in Figs. 3B and 4A. Consequently, our models correctly assigned most α-helical MDPs and most coiled and mixed PBPs from the external datasets (Figs. 4B and 4C). In contrast, most coiled peptides in the external validation were misclassified (Fig. 4B: 13 out of 24, Fig. 4C: 20 out of 24) despite 188 coiled MPPs in the model sets. Both imbalances among structures and classes affected the model performances.

### Mitigating the structural bias by subset selection and data reduction

To tackle the effects imbalanced structural regions have upon the performances of our models, we developed new binary and ternary classifiers that could predict the three mechanisms of action (MDPs, MPPs, and PBPs), invariably from their structural diversity. These models were either trained from specific structural subsets—predicted α-helices (1.1 and 2.1), predominantly coils (1.3 and 2.3), and mixed structures (1.4 and 2.4)—or the structural subsets I, III, and IV in their training sets were balanced out, giving the new training sets V, and the models 1.5 and 2.5. In the latter, we processed by randomly reducing the number of folding sequences to (loose) α-helices from the primary peptide class(es). We repeated the procedure five times; the final classes were selected by majority vote (mode). All three classes were balanced using the ROSE oversampling method. The performances of all models are summarized in Table 2 (binary models 1.1–1.5) and Table 3 (ternary models 2.1–2.5). We added our reference models 1.0 and 2.0 performance metrics for direct comparison.

**Table 2:** Performance of RF binary classifiers to distinguish between membrane-disrupting and membrane-penetrating peptides using 49 sequence-based physicochemical descriptors under the ROSE method.

Model	Structural region(s)	Acc	Prec	Rec	F1	MCC	CK	AUC ROC
1.0	Complete	**88.0**	**90.1**	**0.855**	**0.874**	**0.761**	**0.760**	**0.940**
		**83.3**	**86.4**	**0.781**	**0.820**	**0.669**	**0.666**	**0.832**
1.1	I	95.5	93.8	0.976	0.956	0.911	0.910	0.985
		89.5	88.9	0.727	0.800	0.736	0.730	0.845
1.3	III	90.5	97.7	0.829	0.894	0.819	0.809	0.987
		83.3	85.7	0.857	0.857	0.657	0.657	0.826
1.4	IV	85.0	88.5	0.833	0.841	0.690	0.690	0.911
		92.3	100	0.667	0.800	0.778	0.755	0.833
1.5	V	85.4	86.6	0.920	0.844	0.851	0.708	0.707
		83.2	81.1	0.829	0.811	0.810	0.660	0.658

Performance values of baseline RF models are given in bold.

**Table 3:** Performance of RF ternary classifiers to distinguish between membrane-disrupting and membrane-penetrating and protein-binding peptides using 56 sequence-based physicochemical descriptors under the ROSE method.

Model	Structural region(s)	Acc	Prec	Rec	F1	MCC	CK
2.0	Complete	**86.7**	**87.2**	**0.867**	**0.867**	**0.801**	**0.800**
		**83.5**	**84.6**	**0.833**	**0.836**	**0.753**	**0.750**
2.1	I	93.7	93.8	0.937	0.936	0.905	0.905
		77.0	80.2	0.759	0.755	0.653	0.639
2.3	III	92.1	92.8	0.922	0.920	0.883	0.882
		81.4	79.9	0.827	0.812	0.686	0.684
2.4	IV	97.1	97.5	0.968	0.969	0.955	0.955
		89.3	91.3	0.868	0.877	0.818	0.812
2.5	V	81.8	82.6	0.818	0.816	0.727	0.722
		78.5	79.8	0.782	0.784	0.680	0.674

Performance values of baseline RF models are given in bold.

We noted that most structure-specific models outperformed their unspecific parent classifiers (models 1.0 and 2.0). For example, the α-helix-specific binary classifier (1.1) presented respective training and testing accuracies of 95.5% and 89.5%, far better than model 1.0 with 88.0% and 83.3% accuracy values. This improvement was also observed across the other performance metrics, i.e., precision, recall, F1, MCC, CK, and ROC AUC values. Likewise, the coil-specific binary classifier (1.3) was slightly improved, with training and testing accuracies of 90.5% and 83.3%. On the contrary, model 1.4, trained on a handful of peptide sequences with AF2-predicted mixed structures, demonstrated poorer performance—see Table 2. Looking at model 1.5, removing representative sequences from subset I (AF2-predicted α-helices) at random led to information loss in the training process, translating to lower accuracies of 85.4% and 83.2%. However, the recall indicated stronger sensitivity. The other classification metrics also supported this observation. The structure-specific models 2.1–2.4 outperformed their parent ternary classifier 2.0—see Table 3. Unlike model 1.4, the ternary model 2.4 trained on many PBP sequences with AF2-predicted mixed structures, leading to better classification metrics.

**Figure 5:**
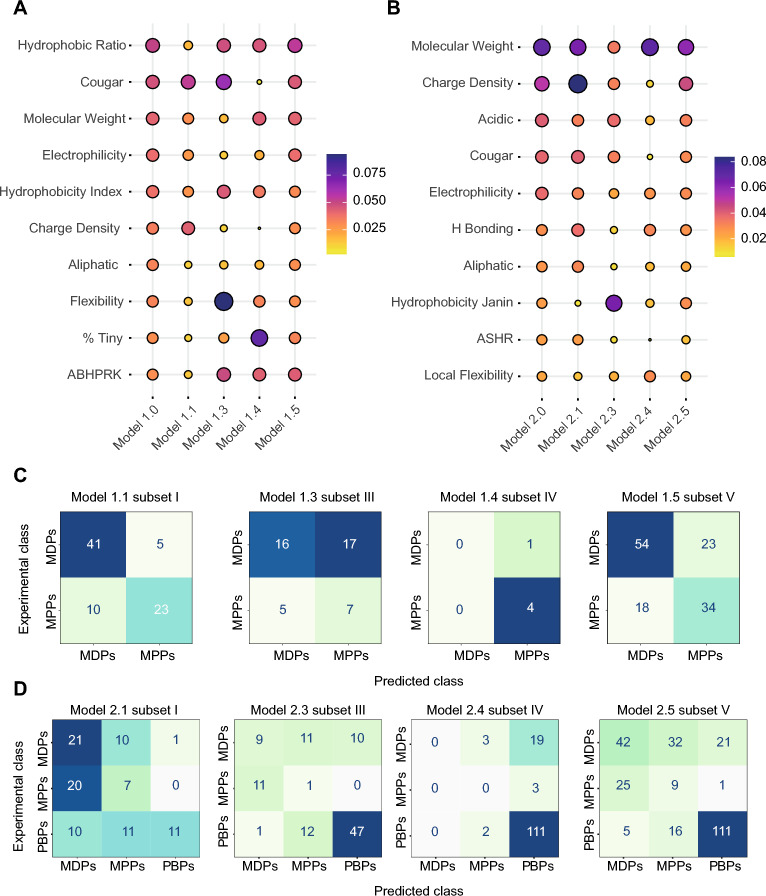
Predictions and feature importances of top-performing structure-aware models. Heatmaps showing the importances of key physicochemical descriptors (rows) in (**A**) our best binary classifiers (1.0–1.5) and (**B**) our best ternary classifiers (2.0–2.5). Confusion matrices of the external validation as a whole and its subsets (I, III, IV), according to binary models 1.1–1.5 (**C**) and ternary models 2.1–2.5 (**D**).

A benefit of building predictive models from random forest and other tree-based algorithms is the built-in estimation of feature importances. We hypothesized that importance scores of physicochemical properties as features would be sensitive to the structural awareness of our models. For example, the critical features involved in classifying α-helical sequences would differ from those classifying coiled or β-stranded membrane-active peptides. We measured the importance scores of 49 physicochemical properties for binary models 1.0–1.5 and 56 properties for ternary models 2.0–2.5 in Tables S8 and S9, respectively. To ease readership, we showed the 10 most common features and their importance scores (colored circles) in Fig. 5A,B.

In Fig. 5A, we recall that hydrophobicity (*hydrophobic ratio*, *H. index*), size (*molecular weight*), amphiphilicity (*aliphatic amino acids*, *% tiny residues*, *flexibility*, *ABHPRK*), and charge (*charge density*, *electrophilicity*) were among these features that distinguish membrane-disrupting peptides (MDPs) from membrane-penetrating peptides (MPPs). With model 1.1, the two global peptide descriptors *cougar* and *charge density* were essential to classify α-helical MDPs and MPPs, with importance scores (IS) of 0.053 and 0.043—Table S8. In contrast, *flexibility* was the main physicochemical property to classify coiled membrane-active peptides (model 1.3—IS_*flexibility*_ 0.094). Finally, hydrophobicity, size, and amphiphilicity played important roles in membrane-active peptides with AF2-predicted mixed structures. Structure-agnostic model 1.5 shared at least the 10 most important features as parent model 1.0 with importance scores in the same order of magnitude, except for the *ABHPRK* property (IS_*ABHPRK*_ 0.030 vs. 0.044, Table S8). Diminishing α-helical sequences in the training process reduced the predictive power of model 1.5, but it did not induce changes in feature importance. In other words, structure-specific models and their feature importance are sensitive to the (partial or complete) proportions of structural subsets (I-IV), supporting our hypothetical statement.

In Fig. 5B, ternary model 2.0 estimated that *molecular weight* and *charge density* were the two most essential descriptors to differentiate MDPs, MPPs, and PBPs, with respective importance scores of 0.069 and 0.052 (Table S9). Other elements related to charge (*electrophilicity*, *hydrogen bonding*, *charge density*), hydrophobicity (*Janin scale*, *ASHR*), and amphiphilicity played minor roles among the three classes. Not surprisingly, *molecular weight* (0.065) and *charge density* (0.084) were crucial for classifying α-helical MDPs, MPPs, and PBPs in model 2.1, with ~ 54% sequences in model 2.0 predicted to adopt α-helices. Hydrophobicity and elements of charge and *molecular weight* (to a lesser degree of importance—0.035) distinguished coiled membrane-active and protein-binding peptides (model 2.3). Finally, size (*molecular weight*) is the leading property that separates membrane-active and protein-affine mixed structures (model 2.4—IS_*MW*_ 0.071). Like model 1.5, structure-agnostic model 2.5 shared the same essential features as its parent model (2.0), such as *molecular weight*, *acidic residues*, and *charge density*, with respective scores of 0.059, 0.047, and 0.039 (Table S9). Unlike model 1.5, removing α-helical sequences in the training process did not induce a loss in predictive power.

We evaluated the performances of the structure-specific models (1.1–1.4, 2.1–2.4) and structure-agnostic models (1.5 and 2.5) against structural subsets of the external validation dataset (I, III, and IV) and its “balanced” form (V), as detailed in Fig. 5C,D. From the confusion matrices, we derived the five key performance indicators—accuracy, precision, recall, specificity, and F1 score, as per Eqs. (S3)–(S6) and (S9), and we compiled the results in Table S10. Overall, our analysis of the external validation subsets revealed three distinct trends in class predictions based on the structure awareness of the models: an increase, a decrease, or no change.

Narrowing the training process to sequences folded into α-helices has improved model 1.1 predictive power, compared to model 1.0 (Table 2). Applying these models to the α-helical external validation subset (I) also increased across all binary classification metrics, indicative of the correct assignment of MDPs and MPPs, i.e., precision, recall, and F1 scores of 0.804, 0.891 and 0.845 (model 1.1—Fig. 5C and Table S10). Similarly, the ternary model 2.4 learned to classify peptides folding into AF2-predicted mixed structures, particularly PAP sequences. Consequently, the many PAP sequences within the external validation subset (IV) were correctly identified with a classification accuracy of 80.4% (model 2.4—Fig. 5D and Table S10). These two examples illustrated that training and external validation subsets share structures and classes to ensure the correct classification of sequences.

Without shared structures and classes, the external validation subsets would score identical metrics or lead to misclassification. For example, the application of binary model 1.4 to subset IV resulted in the same accuracy of 80.0% despite an improved training process to classify AF2-predicted mixed structures (models 1.0 and 1.4—Tables 2 and S10). This result is associated with the quasi-absence of MDP/MPP sequences within the external validation subset (IV); see Figs. 3C and 4A. Likewise, many sequences from subsets (I) and (III) were misclassified, as indicated by the lower accuracies and recall values in Table S10, despite the improved performances of structure-specific models 1.3, 2.1, and 2.3. The other classification metrics (precision, specificity, and F1) vary from the imbalance between structural subsets and classes. As such, model 1.3 to subset (III) incorrectly assigned MDPs (actual positives) and misclassified many MPPs (actual negatives) as MDPs, leading to higher precision, lower specificity, and lower F1 scores from the underrepresented coiled MDPs. Applying model 2.3 to subset (III) struggled to distinguish between MDPs and MPPs classes due to the abundance of coiled PBPs, leading to lower precision, recall, specificity, and F1 score—see Fig. 5D and Table S10. Conversely, the abundance of α-helical MDPs over the other two classes in the subset (I) drove higher precision, specificity, and F1 scores. In other words, few MDPs (positives) were misidentified, so more α-helical sequences from underrepresented MPPs and PBPs could be predicted correctly (model 2.1—Table S10).

Finally, removing random α-helical sequences during training resulted in sub-optimal models 1.5 and 2.5 with similar or reduced performances, see Tables 2 and 3. Applying these models to the balanced subset (V) showed identical or worsened classification metrics (models 1.5 and 2.5—Table S10). In Fig. 3B, removing several α-helical sequences randomly would lead to more balanced yet less informative structural landscapes of MDPs and MPPs. In contrast, mixed structures would dominate among PBPs. Consequently, removing sequences has accentuated class and structural imbalances. Our ternary model 2.5 is less effective at identifying between the three classes and structural subsets (I-IV).

## Discussion

The primary focus of our study aimed to identify critical features that distinguished membrane-active peptides (MAPs) from protein-binding peptides (PBPs). Our research is an extension of two seminal statistical analyses deciphering the relationships between peptides and their behaviors when interacting with cell membranes^34,35^. In the first study, Lee and co-workers developed a sequence-first Support Vector Classifier between antimicrobial peptides (AMPs). The authors identified that their model was good at predicting the membrane activity of AMPs rather than their antimicrobial nature^34,77^. Two years later, Brand and co-workers classified membrane-active peptides by combining differential scanning calorimetry results and circular dichroism experiments using unsupervised learning^35^. Although limited to α-helical AMPs, both original studies uncovered that physicochemical properties (i.e., amphiphilicity, helical propensity) could help predict the peptides’ antimicrobial nature and membrane activity. In our hands, membrane-active peptides presented higher levels of lysine and positively charged dipeptides, suggesting that the peptides primarily interact with negatively charged lipids across cell membranes. Regarding their global physicochemical properties, MAPs were characterized by higher amphiphilicity and net charges than protein-binding peptides, corroborating the findings above. Membrane-disrupting peptides exhibited higher levels of hydrophobicity, correlating with their prolonged time within cell membranes before aggregating and pore formation. In contrast, protein-binding peptides showed more significant indices of penetration depth and a greater tendency to aggregate in vitro.

Encouraged by our initial findings, we developed machine learning classifiers to predict membrane or protein activity (recognition) between the two or three classes. We employed 12 binary classification algorithms to distinguish between MDPs and MPPs and 9 ternary classification algorithms to differentiate between the MAP classes and PBPs. Overall, the Random Forest Classification (RFC) emerged as the most effective algorithm for both binary and ternary classifiers, achieving the highest accuracies (86.7–88.0% for training and 83.3–83.5% for testing) when combined with the oversampling method ROSE. Our study further supported the evidence that tree-based models outperformed other algorithms in classification tasks using tabular data such as *modlAMP* descriptors^49,73–75^ or other features^76^.

Aware of the possible over-representation of α-helices among AMPs^29^, we estimated the structural landscapes of our model datasets, revealing a majority of peptides assuming α-helical and loose structures (subset I—53.9%), two minorly represented coiled structures (III—33.1%) and mixed structures (IV—13.0%), and the complete absence of β-stranded peptides (II). An objective evaluation of the structural awareness demonstrated that our preliminary models were biased toward predicting the likelihood of sequences forming α-helical structures by assigning them high-class probabilities. In other words, our models were structurally biased. Noteworthy, most ML models are sequence-based and have not incorporated structural information in their predictions for peptide design^19–25^. Some researchers have, however, reduced their training sequences to a specific amino acid distribution, inducing involuntary structural constraints. For instance, Dean and co-workers have developed tree-based regression models that predict the minimum inhibitory concentration (MIC) against strains such as *E. coli*, *S. aureus*, and *P. aeruginosa*, specifically for peptides exempted from cysteine and proline that exclusively fold into α-helices^73^. Our study is the first assessment of structural bias in predictive models and of the structural effects upon prediction. Additional independent studies are strongly encouraged.

In the latter part of our study, we tackled the structural bias in our predictions by developing new classification models tailored to specific structural subsets, denoted as structure-specific models 1.1–1.4 and 2.1–2.4. We rectified the datasets by adjusting class proportions with the oversampling method ROSE. Additionally, we explored the impact of reducing the number of sequences from the dominant subset, i.e., loose α-helices (I), leading to structure-agnostic models 1.5 and 2.5. Generally, the structure-specific models surpassed their non-specific parent classifiers (1.0 and 2.0) when the training sets contained abundant sequences folding into those structures. However, it became apparent that the sequences to be tested must adopt the same structures to ensure accurate predictions. Notably, models 1.1 and 2.4 exhibited significant increases in training and testing accuracies compared to models 1.0 and 2.0, with similar improvements observed across other performance metrics. Conversely, the absence of sequences sharing the same structural landscape hindered model performance. Similarly, the random removal of α-helical sequences within the over-represented structure subset (I) exacerbated class and structural imbalances, resulting in underperforming structure-agnostic models. While balancing the training data is essential, it must be done thoughtfully to avoid information loss and compromise the model’s predictive power. In terms of feature importance, our investigation revealed distinct features that played crucial roles in differentiating between structure-specific models. The models built on α-helical sequences prioritized hydrophobicity, size, and charge, whereas those tailored for coiled structures value flexibility. These findings prompt us to reconsider whether the amphiphilic nature is an exclusive characteristic of membrane-active peptides with α-helical folds.

## Conclusion

This study delved into the critical features (*i.e*., dipeptides and global physicochemical properties) distinguishing between membrane-disrupting peptides, membrane-penetrating peptides, and protein-binding peptides, leading to the development of robust machine-learning classifiers. While our focus was on predicting these mechanisms of action, our investigation also unveiled the rapid assessment of structural landscapes in peptide datasets and a structural bias in our initial models. They were inclined to predict accurately the mechanisms of action of sequences forming α-helical structures. To address this bias, we explored two strategies: creating new predictive models trained on specific structural subsets (referred to as structure-specific models) or utilizing structurally balanced datasets (referred to as structure-agnostic models). Overall, the structure-specific models tended to outperform the non-specific ones, particularly when the training and test sequences shared similar structural landscapes. Conversely, removing α-helical sequences randomly worsened the model performances, indicating the need for thoughtful data balancing to avoid information loss. Moreover, our analysis highlighted the sensitivity of important features across these models to the structure classes, underlining the significance of considering structural nuances in peptide classification. This work is one of the first studies to assess structural bias in predictive models and to suggest that structural effects are crucial for accurate predictions, an issue not commonly addressed in sequence-based machine learning models. It is a significant step forward in helping our understanding of the sequence-structure–function relationships and the design of peptide-based therapeutics using artificial intelligence.

### Supplementary Information

Supplementary Information.

## Data Availability

Supporting data in this article are provided in Supporting Information. Data and scripts can be downloaded from the public GitHub repository https://github.com/Puga8Ma/Structure-aware-ML-for-AMP-discovery.
