# Supplementary material for: Structure-aware machine learning strategies for antimicrobial peptide discovery
Source: Sci Rep. 2024 May 25;14:11995. doi: 10.1038/s41598-024-62419-y (PMC11127937; doi:10.1038/s41598-024-62419-y)
Supplement: Supplementary file 1 — Supplementary Information. [file 41598_2024_62419_MOESM1_ESM.docx]

**Supporting Information**

**Equations** 1

**List S1.** 76 selected physicochemical properties from R library *Peptides*^1^. 2

**List S2.** 8 selected physicochemical properties with DBAASP^2^. 3

**List S3.** 33 selected physicochemical properties using Python package *modlAMP*^3^. 4

**Figure S1.** Correlations between 117 physicochemical properties (MDPs vs. MPPs). 6

**Figure S2.** Correlations between 117 physicochemical properties (MDPs vs. MPPs vs. PBPs). 7

**Figure S3.** Statistical pipeline (MDPs vs. MPPs). 8

**Figure S4.** Statistical pipeline (MAPs vs. PBPs). 9

**Figure S5.** Boxplots of 56 physicochemical properties. 10

**Table S1.** Significant physicochemical properties between 2 or 3 classes. 11

**Table S2.** Performances of binary classification algorithms under ROSE oversampling. 18

**Table S3.** Performances of binary classification algorithms under SMOTE oversampling. 19

**Table S4.** Performances of binary classification algorithms under ADASYN oversampling. 20

**Table S5.** Performances of ternary classification algorithms under ROSE oversampling. 21

**Table S6.** Performances of ternary classification algorithms under SMOTE oversampling. 22

**Table S7.** Performances of ternary classification algorithms under ADASYN oversampling. 23

**Table S8.** Feature importance scores of the 49 physicochemical properties used for

the predictive models 1.0-1.5. 24

**Table S9.** Feature importance scores of the 56 physicochemical properties used for

the predictive models 2.0-2.5. 26

**Table S10.** Performances of binary and ternary models based on different levels of

structure awareness. 28

**Equations**

$X = \frac{\left( x - x_{min} \right)}{\left( x_{max} - x_{min} \right)}$ (**Eq.1**)

$X_{validation} = \frac{\left( x_{val} - x_{min} \right)}{\left( x_{max} - x_{min} \right)}$ (**Eq.2**)

$Accuracy = \frac{TP + TN}{TP + TN + FP + FN}$ (**Eq. 3**)

$Precision or PPV = \frac{TP}{TP + FP}$ (**Eq. 4**)

$Recall or TPR or Sensitity = \frac{TP}{TP + FN}$ (**Eq. 5**)

$F1 = 2\times\frac{Precision \times Recall}{Precision + Recall}$ (**Eq. 6**)

$MCC = \frac{TP \times TN - FP \times FN}{\sqrt{(TP + FP) \times(TP +FN) \times(TN + FP) \times(TN +FN)}}$ (**Eq. 7**)

where true positive (TP) is the number of true membrane-disrupting peptides that are predicted correctly; true negative (TN) is the number of true membrane-penetrating peptides that are predicted correctly; false positive (FP) is the number of true membrane-disrupting peptides that are predicted to be membrane-penetrating peptides; true negative (TN) is the number of true membrane-penetrating peptides that are predicted to be membrane-disrupting peptides.

${Cohen's Kappa (CK)} = \frac{P_{O} - P_{E}}{1 - P_{E}}$ (**Eq. 8**)

where P_O_ is the relative observed agreement among raters and P_E_ is the hypothetical probability of chance agreement.

$Specificity or TNR =\frac{TN}{TN + FP}$ ` (**Eq. 9**)

**List S1.** 76 selected physicochemical properties from R library *Peptides*^1^.

**aaComp**, the amino-acid composition of a peptide sequence into 9 categories: Tiny (A + C + G + S + T), Small (A + B + C + D + G + N + P + S + T + V), Aliphatic (A + I + L + V), Aromatic (F + H + W + Y), Non-polar (A + C + F + G + I + L + M + P + V + W + Y), Polar (D + E + H + K + N + Q + R + S + T + Z), Charged (B + D + E + H + K + R + Z), Basic (H + K + R), and Acidic (B + D + E + Z).

**boman**, the Boman potential protein interaction index of a peptide sequence.

**charge**, the net charge of a peptide sequence based on the Henderson-Hasselbach equation.

**hmoment**, the hydrophobic moment of a peptide sequence proposed by Eisenberg^4^.

**hydrophobicity**, the GRAVY hydrophobicity index of a peptide sequence using each time one of the following 38 scales: "Aboderin", "AbrahamLeo", "Argos", "BlackMould", "BullBreese", "Casari", "Chothia", "Cid", "Cowan3.4", "Cowan7.5", "Eisenberg", "Engelman", "Fasman", "Fauchere", "Goldsack", "Guy", "HoppWoods", "Janin", "Jones", "Juretic", "Kidera", "Kuhn", "KyteDoolittle", "Levitt", "Manavalan", "Miyazawa", "Parker", "Ponnuswamy", "Prabhakaran", "Rao", "Rose", "Roseman", "Sweet", "Tanford", "Welling", "Wilson", "Wolfenden", "Zimmerman"

**instaIndex**, the instability of a peptide sequence.

**lengthpep**, the total length of a peptide sequence.

**mw**, the molecular weight [g/mol] of a peptide sequence.

**pI,** the isoelectric point of a peptide sequence.

Four properties derived from all the amino acids in the corresponding peptide sequence using factor analysis scale of generalized amino acid information (FASGAI)^5^:

**F3: Bulky properties**

**F4: Compositional characteristic index**

**F5: Local flexibility**

**F6: Electronic properties (2)**

Five properties derived from Z-scales based on the physicochemical properties of the AAs, including nuclear magnetic resonance spectroscopy and thin layer data chromatography data.

**Z1: Lipophilicity**

**Z2: Steric properties (Steric bulk/Polarizability)**

**Z3: Electronic properties (1) (Polarity / Charge)**

**Z4 and Z5: They relate electronegativity, heat of formation, electrophilicity and hardness.**

Two Cruciani^6^ properties calculated the interaction of each amino acid residue with several chemical groups, such as charged ions, methyl, hydroxyl groups:

**PP2: Hydrophobicity (CP)**

**PP3: H-bonding**

**List S2.** 8 selected physicochemical properties with DBAASP^2^.

**Amphiphilicity index**, the amphiphilicity indices of each amino acid in a peptide sequence divided by the peptide length.

**ASHR**, angles subtended by the hydrophobic residues, measured using the helical wheel representation of the peptide sequence in the ideal α-helix approximation.

**Disorder conformation propensity,** capacity of a peptide to disorder in an aqueous media.

**Linear moment,** the linear moment of a peptide sequence.

**In vitro aggregation:** the ability of a peptide sequence to aggregate based on the physicochemical principles of β-sheet formation (TANGO).

**Penetration depth**, the distance between the geometrical center of the peptide and the membrane.

**Propensity to PPII coil,** refers to a coil structure with a degree of local order akin to the PPII helix, with short lengths interspersed with turns**.**

**Tilt angle,** orientation of the peptide sequence relative to the surface of a membrane.

**List S3.** 33 selected physicochemical properties using Python package *modlAMP*^3^.

**Charge density**, the charge density of a peptide sequence.

**Instability Index**, the instability of a peptide sequence based on the amino acid composition.

**Aromaticity**, the aromaticity of a peptide sequence.

**Aliphatic index**, the thermal stability based on the relative volume filled by aliphatic residues

**Boman index**, the Boman potential protein interaction index of a peptide sequence.

**Hydrophobic ratio**, the hydrophobic ratio of a peptide sequence.

**AASI**, an amino acid selectivity index scale for helical antimicrobial peptides^7^.

**ABHPRK**, features as Acidic, Basic, Hydrophobic, Polar, aRomatic, Kink-inducer.

**Argos**, compute hydrophobicity with the scale of Argos^3^.

**Bulkiness**, amino acid side chain bulkiness.

**Charge phys**, amino acid charge at pH 7.0 (histidine charge +0.1).

**Charge acid**, amino acid charge at acidic pH (histidine charge +1.0).

**Cougar**, selection of global peptide descriptors in *modlAMP.*

**Eisenberg**, the hydrophobicity of a peptide sequence proposed by Eisenberg^4^.

**Ez**, the potential that assesses energies of insertion of amino acid side chains into lipid bilayers.

**Flexibility**, amino acid side chain flexibility scale.

**Grantham**, amino acid side chain composition, polarity and molecular volume.

**Gravy**, the hydrophobicity of a peptide sequence using the GRAVY scale.

**ISAECI,** Isotropic Surface Area (ISA) and Electronic Charge Index (ECI) of amino acids^8^.

**Janin**, the hydrophobicity of a peptide sequence using the Janin scale.

**Kyte Doolittle**, the hydrophobicity of a peptide sequence using the Kyte Doolittle scale.

**Hopp-Woods**, the hydrophobicity of a peptide sequence using the Hopp-Woods scale.

**Levitt alpha ^c^,**  alpha-helix propensity using the Levitt scale.

**MSS**, a graph-theoretical index that reflects topological shape and size of AA side chains^9^.

**MSW**, the scale derived from the molecular surface based WHIM descriptor (MS-WHIM)^10^.

**PEPCATS,** pharmacophoric features based on PEPCATS scale.

**Polarity**, the polarity of a peptide sequence.

**PPCALI**, 143 amino acid properties derived from a PCA scale^11^.

**Refractivity**, the refractivity index of a peptide sequence**.**

**t scale**, 6 different scales T1-T6 of the GRID program^12^.

**TM tend**, amino acid transmembrane propensity scale^13^.

**Z1-Z3**, three amino acid scales interpreted as reflecting lipophilicity (z1), steric properties (z2), and electronic properties (z3)^14^.

**Z4-Z5**, two additional amino acid scales related to electronegativity, heat of formation, electrophilicity and hardness^15^.

**
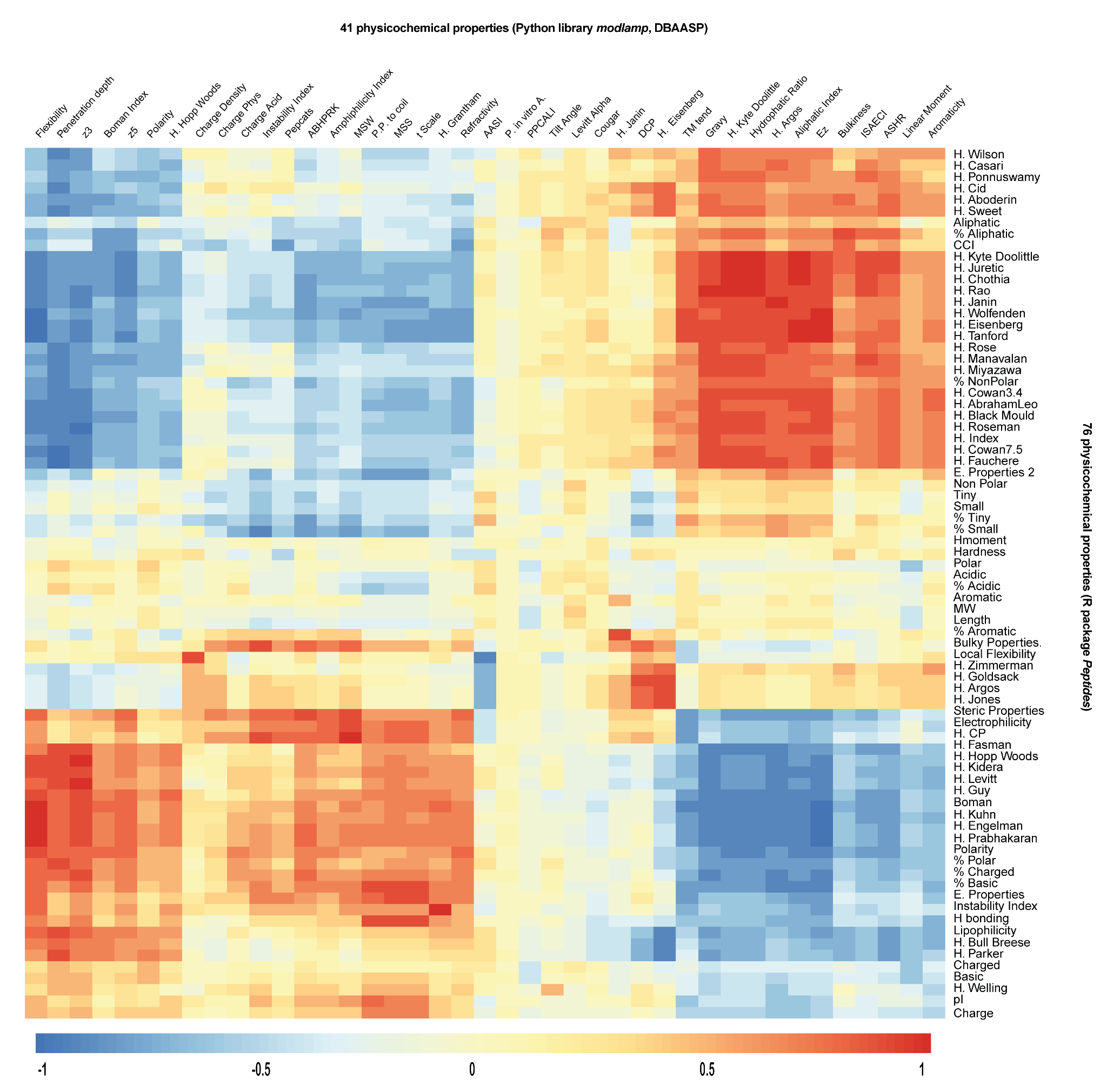
**

**Figure S1** Correlations between 117 physicochemical properties from 749 sequences (MDPs vs. MPPs).

**
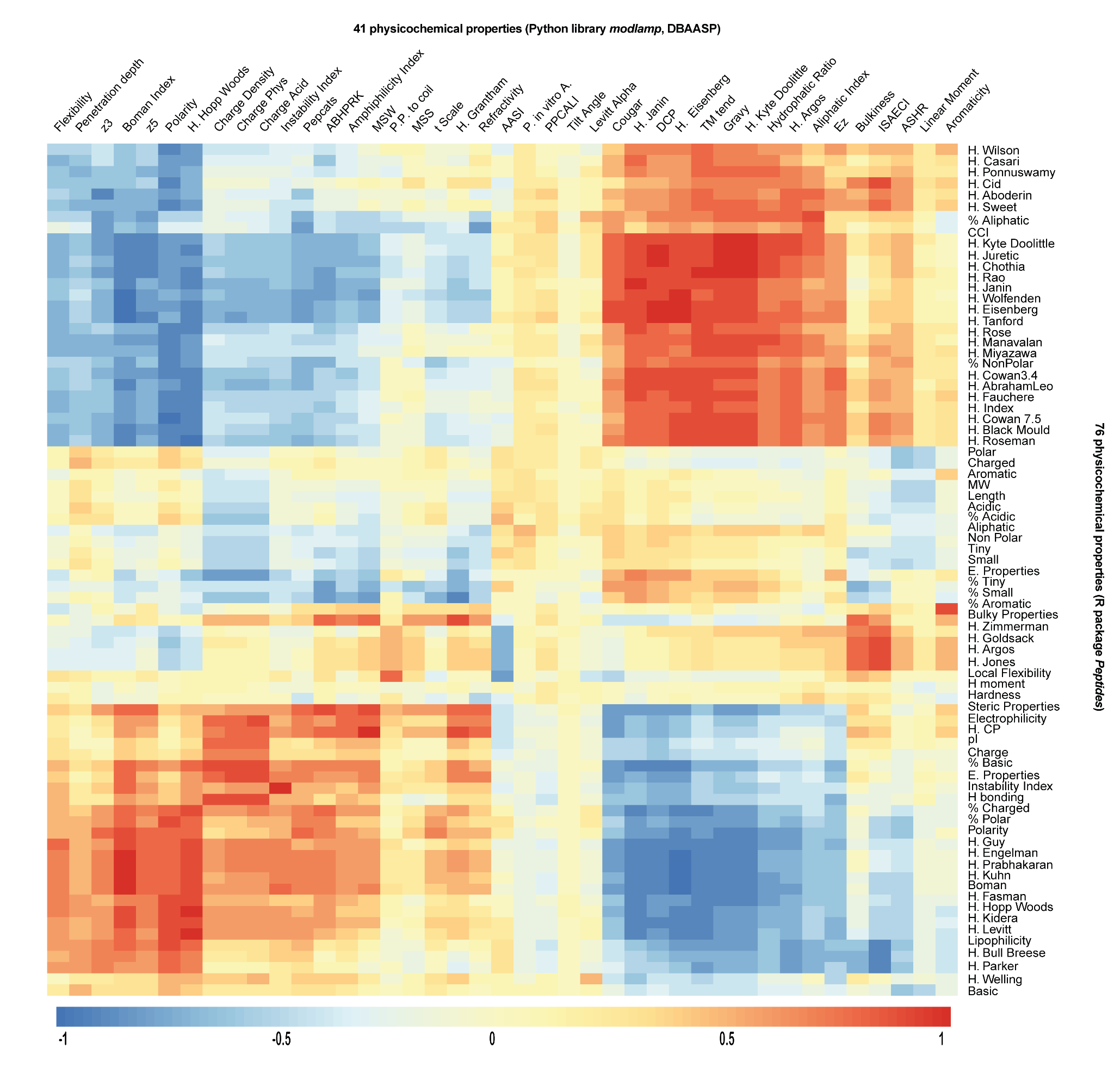
**

**Figure S2** Correlations between 117 physicochemical properties from 1057 sequences (MDPs vs. MPPs vs. PBPs).

**
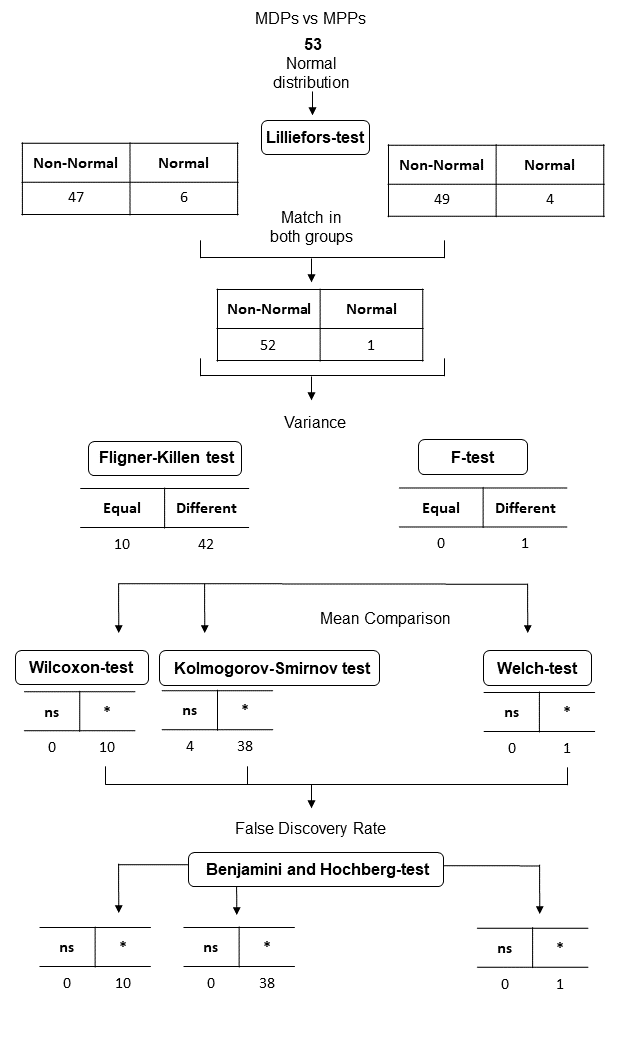
**

**Figure S3.** Statistical pipeline (MDPs vs. MPPs).

**
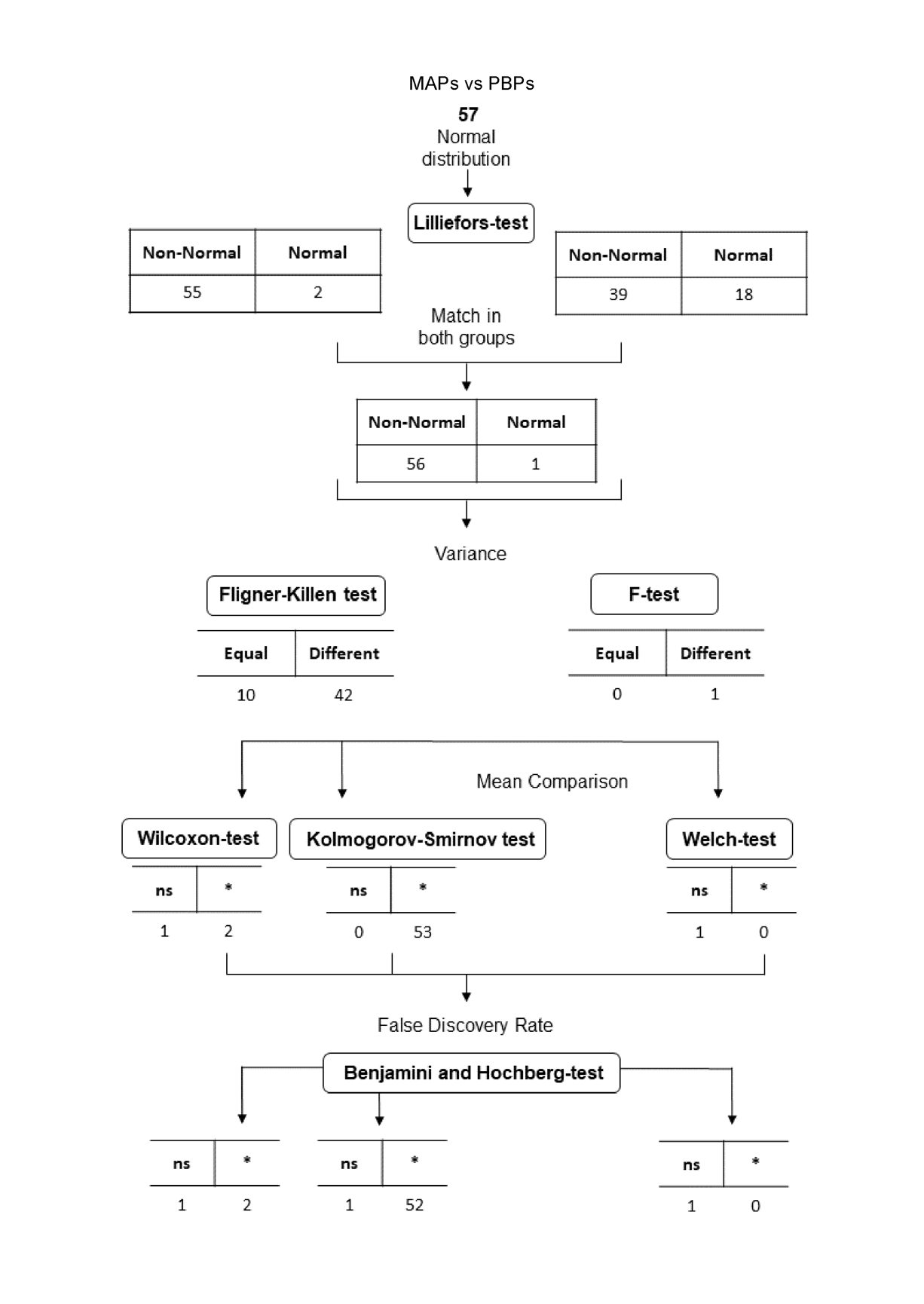
**

**Figure S4.** Statistical pipeline (MAPs vs. PBPs).

**Table S1.** Significant physicochemical properties between 2 or 3 classes.

| **Property** | **MDPs: MPPs** | | **MAPs : PBPs** | |
| --- | --- | --- | --- | --- |
|  | *Test* | *P-value* | *Test* | *P-value* |
| 1. H moment | Kolmogorov-Smirnov | 9.21E-06 | - | ns |
| 2. H. Wilson | Wilcoxon | 2.57E-08 | - | ns |
| 3. Aromatic | - | ns | Kolmogorov-Smirnov | 0 |
| 4. Basic | - | ns | Kolmogorov-Smirnov | 1.23E-09 |
| 5. % Aromatic | - | ns | Kolmogorov-Smirnov | 2.10E-05 |
| 6. % Charged | - | ns | Kolmogorov-Smirnov | 1.78E-07 |
| 7. Linear Moment | - | ns | Wilcoxon | 2.20E-14 |
| 8. H. Wolfenden | - | ns | Kolmogorov-Smirnov | 7.79E-12 |
| 9. H. Janin | - | ns | Kolmogorov-Smirnov | 2.62E-11 |
| 10. pI | - | ns | Kolmogorov-Smirnov | 0 |
| 11. H. Cid | - | ns | Kolmogorov-Smirnov | 8.43E-10 |
| 12. H. Jones | Kolmogorov-Smirnov | 0.00143 | Kolmogorov-Smirnov | 2.22E-16 |
| 13. H. Ponnuswamy | Kolmogorov-Smirnov | 0 | Kolmogorov-Smirnov | 7.55E-06 |
| 14. H. Welling | Kolmogorov-Smirnov | 2.45E-10 | Kolmogorov-Smirnov | 1.08E-06 |
| 15. H. Zimmerman | Kolmogorov-Smirnov | 1.60E-05 | Kolmogorov-Smirnov | 1.37E-10 |
| 16. Charge | Wilcoxon | 4.95E-08 | Kolmogorov-Smirnov | 0 |
| 17. MW | Wilcoxon | 9.06E-07 | Kolmogorov-Smirnov | 0 |
| 18. Aliphatic | Wilcoxon | 2.47E-27 | Kolmogorov-Smirnov | 0 |
| 19. Acidic | Kolmogorov-Smirnov | 5.03E-05 | Kolmogorov-Smirnov | 0 |
| 20. % Tiny | Kolmogorov-Smirnov | 0 | Kolmogorov-Smirnov | 1.20E-10 |
| 21. % Small | Kolmogorov-Smirnov | 2.44E-06 | Kolmogorov-Smirnov | 3.35E-09 |
| 22. % Aliphatic | Kolmogorov-Smirnov | 0 | Kolmogorov-Smirnov | 2.38E-06 |
| 23. % Polar | Kolmogorov-Smirnov | 2.57E-11 | Kolmogorov-Smirnov | 3.06E-10 |
| 24. % Acidic | Kolmogorov-Smirnov | 3.20E-05 | Kolmogorov-Smirnov | 0 |
| 25. Electronic Properties | Kolmogorov-Smirnov | 3.55E-08 | Kolmogorov-Smirnov | 1.44E-15 |
| 26. Electrophilicity | Kolmogorov-Smirnov | 0 | Kolmogorov-Smirnov | 0 |
| 27. Hardness | Kolmogorov-Smirnov | 1.98E-05 | Kolmogorov-Smirnov | 0.00107 |
| 28. Hydrogen bonding | Kolmogorov-Smirnov | 3.07E-08 | Kolmogorov-Smirnov | 0 |

ns = not significant.

**Table S1 (suite).** Significant physicochemical properties between 2 or 3 classes.

| **Property** | **MDPs: MPPs** | | **MAPs : PBPs** | |
| --- | --- | --- | --- | --- |
|  | *Test* | *P-value* | *Test* | *P-value* |
| 29. H. index | Kolmogorov-Smirnov | 0 | Kolmogorov-Smirnov | 6.71E-05 |
| 30. Bulky properties | Wilcoxon | 4.45E-15 | Kolmogorov-Smirnov | 1.22E-15 |
| 31. CCI | Wilcoxon | 2.25E-15 | Kolmogorov-Smirnov | 1.98E-05 |
| 32. Local flexibility | Kolmogorov-Smirnov | 1.95E-07 | Kolmogorov-Smirnov | 4.33E-12 |
| 33. E. Properties 2 | Kolmogorov-Smirnov | 4.44E-16 | Kolmogorov-Smirnov | 0 |
| 34. Penetration depth | Wilcoxon | 5.42E-16 | Wilcoxon | 7.48E-07 |
| 35. P. in vitro A. | Kolmogorov-Smirnov | 0 | Kolmogorov-Smirnov | 0 |
| 36. ASHR | Kolmogorov-Smirnov | 0.000238 | Kolmogorov-Smirnov | 0 |
| 37. Amphiphilicity | Kolmogorov-Smirnov | 0 | Kolmogorov-Smirnov | 2.22E-16 |
| 38. P. to P. coil | Kolmogorov-Smirnov | 1.15E-09 | Kolmogorov-Smirnov | 1.91E-10 |
| 39. Charge Density | Kolmogorov-Smirnov | 0 | Kolmogorov-Smirnov | 0 |
| 40. Instability Index | Kolmogorov-Smirnov | 0 | Kolmogorov-Smirnov | 4.96E-08 |
| 41. Aromaticity | Kolmogorov-Smirnov | 0.00159 | Kolmogorov-Smirnov | 0.000586 |
| 42. Hydrophatic Ratio | Kolmogorov-Smirnov | 0 | Kolmogorov-Smirnov | 4.51E-07 |
| 43. AASI | Kolmogorov-Smirnov | 5.96E-06 | Kolmogorov-Smirnov | 6.58E-14 |
| 44. ABHPRK | Kolmogorov-Smirnov | 0 | Kolmogorov-Smirnov | 6.28E-07 |
| 45. H. Argos | Kolmogorov-Smirnov | 0 | Kolmogorov-Smirnov | 1.33E-05 |
| 46. Bulkiness | Kolmogorov-Smirnov | 1.42E-06 | Kolmogorov-Smirnov | 2.88E-07 |
| 47. Cougar | Kolmogorov-Smirnov | 0 | Kolmogorov-Smirnov | 0 |
| 48. Ez | Kolmogorov-Smirnov | 6.49E-10 | Kolmogorov-Smirnov | 1.08E-14 |
| 49. Flexibility | Kolmogorov-Smirnov | 0 | Kolmogorov-Smirnov | 4.63E-09 |
| 50. Levitt Alpha | Kolmogorov-Smirnov | 2.22E-08 | Kolmogorov-Smirnov | 3.38E-11 |
| 51. MSS | Wilcoxon | 1.08E-09 | Kolmogorov-Smirnov | 1.27E-09 |
| 52. PEPCATS | Kolmogorov-Smirnov | 0 | Kolmogorov-Smirnov | 1.24E-05 |
| 53. PPCALI | Kolmogorov-Smirnov | 2.18E-10 | Kolmogorov-Smirnov | 2.33E-15 |
| 54. Refractivity | Wilcoxon | 4.04E-12 | Kolmogorov-Smirnov | 4.44E-16 |
| 55. t Scale | Kolmogorov-Smirnov | 0.000262 | Kolmogorov-Smirnov | 2.07E-11 |
| 56. z3 | Welch | 5.80E-25 | Kolmogorov-Smirnov | 7.23E-10 |

**
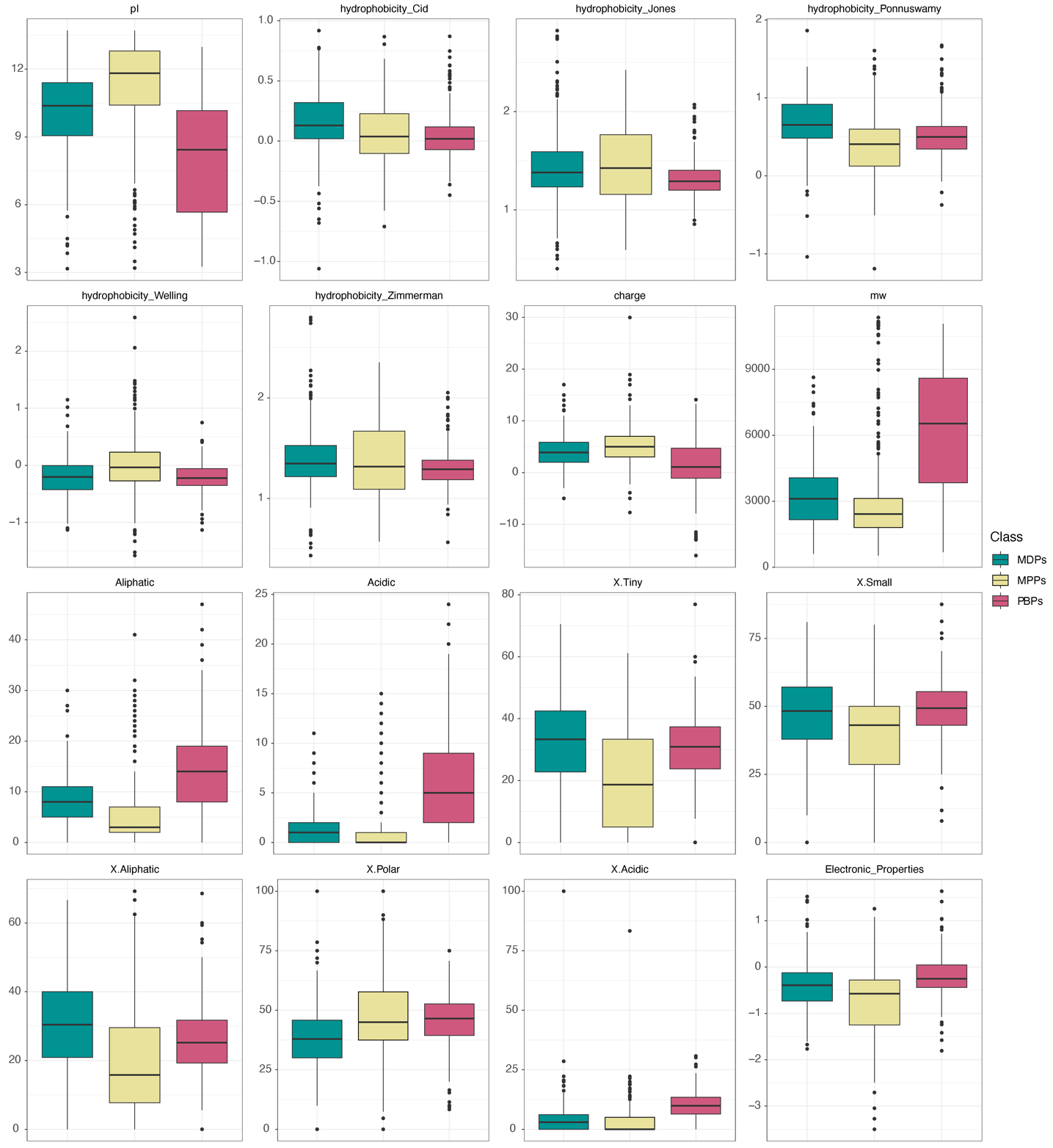
**

**Figure S5** Boxplots of 56 physicochemical properties (1-16).

**
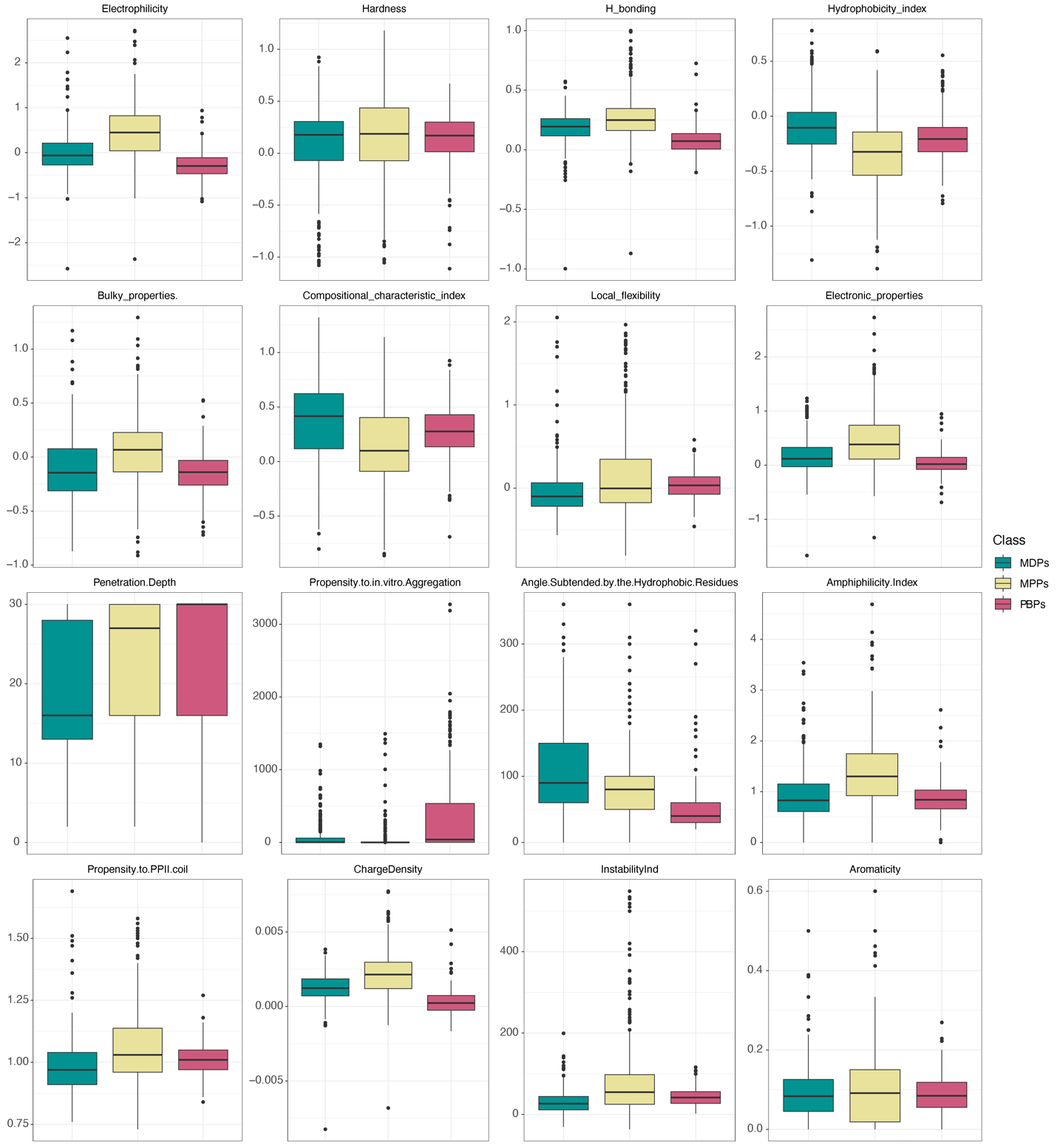
**

**Figure S5 (suite)** Boxplots of 56 physicochemical properties (17-32).

**
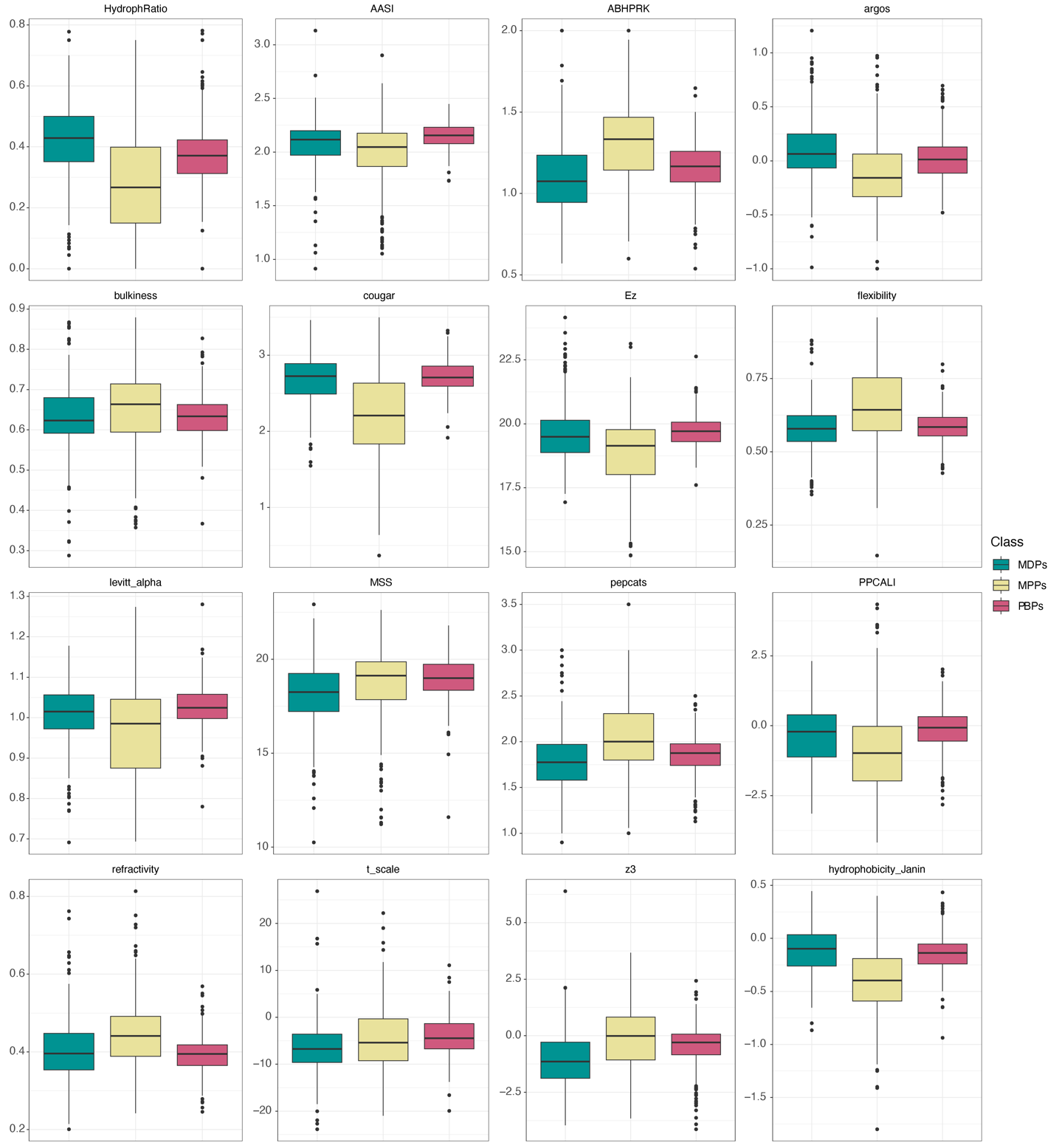
**

**Figure S5 (suite)** Boxplots of 56 physicochemical properties (33-48).

**
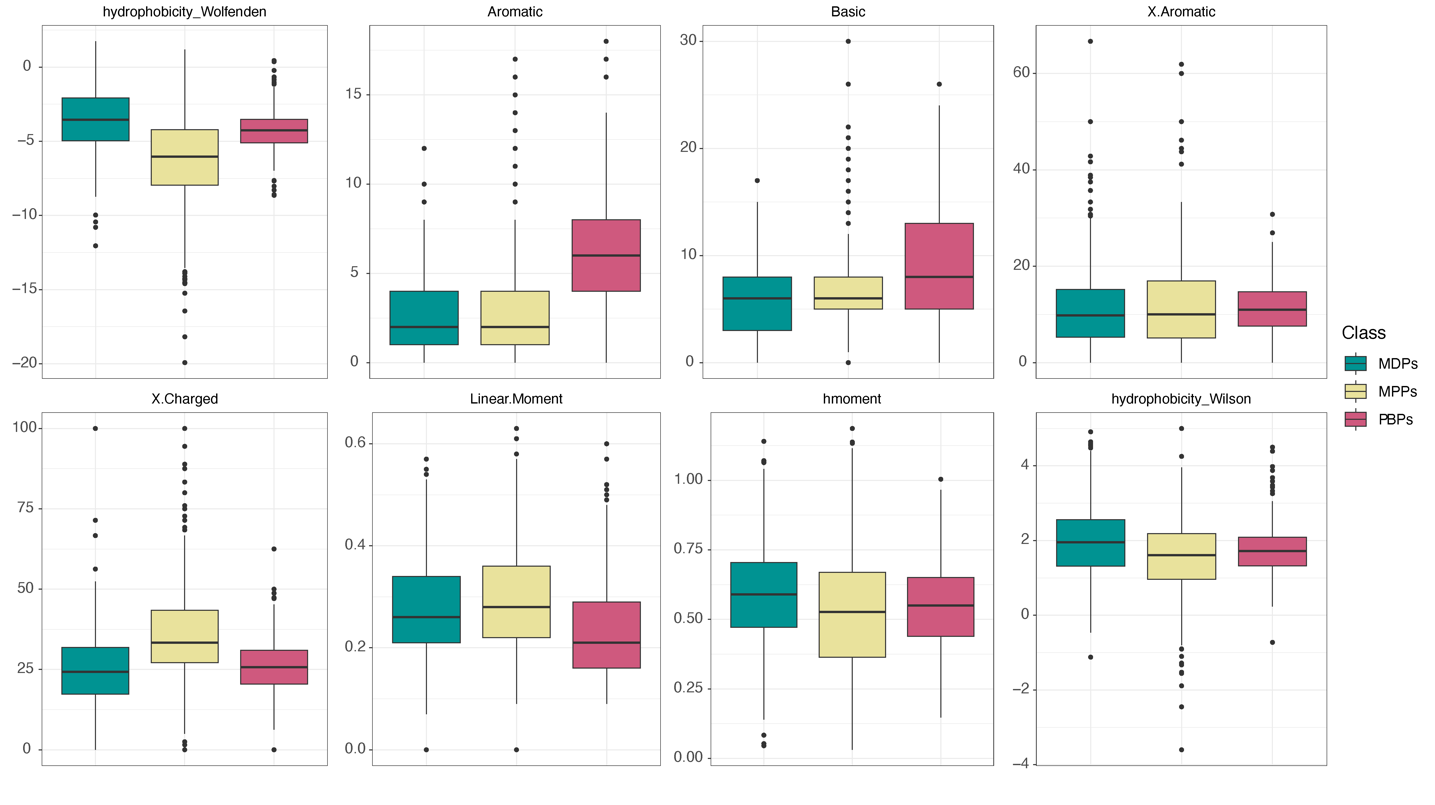
**

**Figure S5 (suite)** Boxplots of 56 physicochemical properties (49-56).

**Table S2.** Performance of 12 binary classifiers to predict membrane-disrupting peptides (MDPs) versus membrane-penetrating peptides (MPPs) using 49 physicochemical properties and ROSE oversampling.

| **Classifier** | **Accuracy** | **Precision** | **Recall** | **F1** | **MCC** | **CK** | **AUC ROC** |
| --- | --- | --- | --- | --- | --- | --- | --- |
| **RFC** | **88.0** | **90.0** | **85.5** | **0.873** | **0.761** | **0.760** | **0.939** |
|  | **83.3** | **86.3** | **78.0** | **0.820** | **0.668** | **0.665** | **0.831** |
| **GBC** | 86.7 | 88.0 | 85.1 | 0.863 | 0.734 | 0.733 | 0.916 |
|  | 85.3 | 85.9 | 83.5 | 0.847 | 0.706 | 0.706 | 0.852 |
| **ABC** | 83.4 | 85.0 | 81.6 | 0.828 | 0.669 | 0.668 | 0.884 |
|  | 78.6 | 78.8 | 76.7 | 0.777 | 0.572 | 0.572 | 0.786 |
| **LDA** | 77.5 | 80.1 | 72.1 | 0.760 | 0.553 | 0.550 | 0.846 |
|  | 76.0 | 81.3 | 65.7 | 0.727 | 0.526 | 0.517 | 0.757 |
| **LR** | 78.7 | 82.0 | 73.7 | 0.774 | 0.576 | 0.573 | 0.842 |
|  | 82.0 | 84.8 | 76.7 | 0.805 | 0.641 | 0.638 | 0.818 |
| **DT** | 82.1 | 80.3 | 85.4 | 0.824 | 0.643 | 0.642 | 0.821 |
|  | 77.3 | 78.2 | 73.9 | 0.760 | 0546 | 0.545 | 0.772 |
| **KNN** | 84.4 | 90.8 | 77.2 | 0.831 | 0.696 | 0.689 | 0.905 |
|  | 79.3 | 80.8 | 72.6 | 0.773 | 0.589 | 0.585 | 0.791 |
| **GNB** | 78.2 | 85.7 | 68.3 | 0.757 | 0.576 | 0.565 | 0.844 |
|  | 78.0 | 85.7 | 65.7 | 0.744 | 0.572 | 0.557 | 0.776 |
| **SVC**  kernel: RBF | 82.7 | 89.6 | 74.2 | 0.810 | 0.663 | 0.653 | 0.899 |
|  | 83.3 | 87.5 | 76.7 | 0.817 | 0.670 | 0.665 | 0.831 |
| **SVC**  kernel: linear | 78.9 | 83.0 | 73.0 | 0.775 | 0.583 | 0.579 | 0.846 |
|  | 80.0 | 86.4 | 69.8 | 0.772 | 0.608 | 0.597 | 0.797 |
| **SVC**  kernel: polynomial | 85.2 | 89.1 | 80.4 | 0.844 | 0.707 | 0.704 | 0.895 |
|  | 83.3 | 90 | 73.9 | 0.812 | 0.675 | 0.664 | 0.830 |
| **SVC**  kernel: sigmoid | 54.1 | 42.7 | 71.2 | 0.532 | 0.087 | 0.082 | 0.762 |
|  | 60.0 | 55.3 | 91.8 | 0.690 | 0.274 | 0.212 | 0.608 |

RF: random forest, GBC: gradient boosting classifier, ABC: AdaBoost classifier, LDA: linear discriminant analysis, LR: logistic regression, DT: decision tree, K-NN: K-nearest neighbor, GNB: Gaussian Naïve Bayes, SVC: support vector machine [RBF: radial basis function]. For all algorithms, the performances on cross-validated model dataset (top lines) and those on the external validation set (bottom lines). Accuracy, precision and recall are expressed in percentages.

**Table S3.** Performance of 12 binary classifiers to predict membrane-disrupting peptides (MDPs) versus membrane-penetrating peptides (MPPs) using 49 physicochemical properties and SMOTE oversampling.

| **Classifier** | **Accuracy** | **Precision** | **Recall** | **F1** | **MCC** | **CK** | **AUC ROC** |
| --- | --- | --- | --- | --- | --- | --- | --- |
| **RFC** | **86.5** | **88.9** | **83.7** | **0.860** | **0.732** | **0.731** | **0.934** |
|  | **83.3** | **84.3** | **80.8** | **0.825** | **0.667** | **0.666** | **0.833** |
| **GBC** | 85.7 | 87.4 | 83.7 | 0.854 | 0.714 | 0.713 | 0.913 |
|  | 84.7 | 85.7 | 82.2 | 0.839 | 0.693 | 0.693 | 0.846 |
| **ABC** | 80.9 | 83.8 | 76.9 | 0.800 | 0.620 | 0.618 | 0.867 |
|  | 83.3 | 83.3 | 82.2 | 0.828 | 0.666 | 0.666 | 0.833 |
| **LDA** | 77.1 | 80.4 | 71.9 | 0.756 | 0.544 | 0.541 | 0.846 |
|  | 77.3 | 83.1 | 67.1 | 0.742 | 0.554 | 0.544 | 0.771 |
| **LR** | 76.5 | 80.7 | 70.1 | 0.747 | 0.534 | 0.530 | 0.841 |
|  | 82.0 | 84.8 | 76.7 | 0.806 | 0.642 | 0.639 | 0.819 |
| **DT** | 77.5 | 77.1 | 79.6 | 0.781 | 0.551 | 0.550 | 0.775 |
|  | 74.0 | 75.0 | 69.9 | 0.723 | 0.480 | 0.479 | 0.739 |
| **KNN** | 83.7 | 87.2 | 79.3 | 0.829 | 0.677 | 0.675 | 0.914 |
|  | 81.3 | 88.1 | 71.2 | 0.788 | 0.636 | 0.625 | 0.811 |
| **GNB** | 76.3 | 84.7 | 64.8 | 0.731 | 0.541 | 0.527 | 0.822 |
|  | 78.7 | 86.0 | 67.1 | 0.754 | 0.584 | 0.571 | 0.784 |
| **SVC**  kernel: RBF | 81.2 | 88.3 | 72.5 | 0.794 | 0.634 | 0.624 | 0.891 |
|  | 82.0 | 85.9 | 75.3 | 0.803 | 0.643 | 0.639 | 0.818 |
| **SVC**  kernel: linear | 78.7 | 83.8 | 71.6 | 0.768 | 0.580 | 0.574 | 0.853 |
|  | 82.0 | 88.3 | 72.6 | 0.767 | 0.648 | 0.638 | 0.818 |
| **SVC**  kernel: polynomial | 84.0 | 87.6 | 79.6 | 0.832 | 0.683 | 0.680 | 0.895 |
|  | 84.0 | 90.2 | 75.3 | 0.821 | 0.687 | 0.678 | 0.838 |
| **SVC**  kernel: sigmoid | 53.8 | 42.4 | 72.4 | 0.533 | 0.083 | 0.077 | 0.752 |
|  | 60.0 | 55.4 | 91.8 | 0.691 | 0.274 | 0.213 | 0.608 |

**Table S4.** Performance of 12 binary classifiers to predict membrane-disrupting peptides (MDPs) versus membrane-penetrating peptides (MPPs) using 49 physicochemical properties and ADASYN oversampling.

| **Classifier** | **Accuracy** | **Precision** | **Recall** | **F1** | **MCC** | **CK** | **AUC ROC** |
| --- | --- | --- | --- | --- | --- | --- | --- |
| **RFC** | **86.5** | **86.9** | **86.0** | **0.864** | **0.730** | **0.730** | **0.934** |
|  | **84.0** | **84.5** | **82.2** | **0.833** | **0.680** | **0.680** | **0.840** |
| **GBC** | 82.4 | 83.7 | 80.7 | 0.819 | 0.648 | 0.647 | 0.898 |
|  | 84.0 | 83.6 | 83.6 | 0.836 | 0.680 | 0.680 | 0.840 |
| **ABC** | 77.0 | 83.6 | 83.6 | 0.836 | 0.680 | 0.680 | 0.840 |
|  | 81.3 | 82.6 | 78.1 | 0.803 | 0.627 | 0.626 | 0.812 |
| **LDA** | 73.6 | 76.9 | 67.7 | 0.713 | 0.476 | 0.472 | 0.811 |
|  | 78.0 | 81.3 | 71.2 | 0.759 | 0.562 | 0.558 | 0.778 |
| **LR** | 74.4 | 78.3 | 67.4 | 0.718 | 0.492 | 0.487 | 0.807 |
|  | 82.0 | 84.8 | 76.7 | 0.806 | 0.642 | 0.639 | 0.819 |
| **DT** | 78.4 | 77.6 | 80.4 | 0.788 | 0.568 | 0.567 | 0.784 |
|  | 74.7 | 72.7 | 76.7 | 0.747 | 0.494 | 0.494 | 0.747 |
| **KNN** | 82.5 | 85.1 | 79.2 | 0.816 | 0.652 | 0.650 | 0.902 |
|  | 81.3 | 82.6 | 78.1 | 0.803 | 0.627 | 0.626 | 0.812 |
| **GNB** | 72.3 | 79.9 | 58.2 | 0.660 | 0.464 | 0.446 | 0.788 |
|  | 79.3 | 86.2 | 68.5 | 0.763 | 0.596 | 0.584 | 0.791 |
| **SVC**  kernel: RBF | 78.8 | 83.7 | 71.2 | 0.763 | 0.583 | 0.576 | 0.865 |
|  | 84.7 | 86.8 | 80.8 | 0.837 | 0.694 | 0.693 | 0.846 |
| **SVC**  kernel: linear | 74.8 | 79.9 | 66.8 | 0.721 | 0.503 | 0.496 | 0.812 |
|  | 80.7 | 83.3 | 75.3 | 0.791 | 0.615 | 0.612 | 0.805 |
| **SVC**  kernel: polynomial | 81.6 | 84.6 | 77.2 | 0.804 | 0.635 | 0.633 | 0.887 |
|  | 85.3 | 89.2 | 79.5 | 0.841 | 0.710 | 0.706 | 0.852 |
| **SVC**  kernel: sigmoid | 51.6 | 16.0 | 25.2 | 0.195 | 0.036 | 0.030 | 0.718 |
|  | 51.3 | 0.0 | 0.0 | 0.000 | 0.000 | 0.000 | 0.050 |

**Table S5.** Performance of 9 ternary classifiers to predict peptide sequences that can disrupt microbial membranes (MDPs), solely penetrate these membranes (MPPs) to reach intracellular targets or present affinity to larger proteins (PBPs) using 56 physicochemical properties and ROSE oversampling.

| **Classifier** | **Accuracy** | **Precision** | **Recall** | **F1** | **MCC** | **CK** |
| --- | --- | --- | --- | --- | --- | --- |
| **RFC** | **86.7** | **87.1** | **0.866** | **0.866** | **0.800** | **0.800** |
|  | **83.5** | **84.6** | **0.832** | **0.835** | **0.752** | **0.749** |
| **ETC** | 78.7 | 78.9 | 0.787 | 0.784 | 0.680 | 0.680 |
|  | 70.3 | 70.2 | 0.701 | 0.701 | 0.551 | 0.551 |
| **GBC** | 85.5 | 86.1 | 0.854 | 0.855 | 0.782 | 0.782 |
|  | 79.7 | 80.0 | 0.798 | 0.796 | 0.696 | 0.694 |
| **DT** | 77.9 | 78.5 | 0.780 | 0.777 | 0.670 | 0.669 |
|  | 68.4 | 68.6 | 0.693 | 0.684 | 0.531 | 0.527 |
| **KNN** | 78.3 | 79.0 | 0.783 | 0.782 | 0.677 | 0.675 |
|  | 76.8 | 78.1 | 0.766 | 0.769 | 0.652 | 0.694 |
| **RNC** | 33.6 | 34.8 | 0.339 | 0.293 | 0.004 | 0.004 |
|  | 33.5 | 35.3 | 0.346 | 0.347 | 0.002 | 0.002 |
| **GNB** | 71.2 | 72.4 | 0.712 | 0.709 | 0.573 | 0.569 |
|  | 74.0 | 74.6 | 0.742 | 0.739 | 0.611 | 0.608 |
| **MNB** | 67.8 | 69.5 | 0.678 | 0.678 | 0.512 | 0.518 |
|  | 75.4 | 76.2 | 0.752 | 0.754 | 0.629 | 0.627 |
| **LDA** | 74.0 | 75.2 | 0.740 | 0.739 | 0.615 | 0.611 |
|  | 75.0 | 75.1 | 0.751 | 0.747 | 0.625 | 0.622 |

RFC: random forest classifier, ETC: extra tree classifier, GBC: gradient boosting classifier, DT: decision tree classifier, KNN: K-nearest neighbor, RNC: radius neighbors classifier, GNB: Gaussian Naïve Bayes, MNB: multinomial Naïve Bayes, LDA: linear discriminant analysis. For all algorithms, the performances on the cross-validated model dataset (top lines) and those on the external validation set (bottom lines).

**Table S6.** Performance of 9 ternary classifiers to predict peptide sequences that can disrupt microbial membranes (MDPs), solely penetrate these membranes (MPPs) to reach intracellular targets or present affinity to larger proteins (PBPs) using 56 physicochemical properties and SMOTE oversampling.

| **Classifier** | **Accuracy** | **Precision** | **Recall** | **F1** | **MCC** | **CK** |
| --- | --- | --- | --- | --- | --- | --- |
| **RFC** | **84.0** | **84.4** | **0.840** | **0.839** | **0.760** | **0.759** |
|  | **84.0** | **84.5** | **0.840** | **0.840** | **0.759** | **0.757** |
| **ETC** | 71.4 | 71.6 | 0.714 | 0.712 | 0.570 | 0.570 |
|  | 70.8 | 71.5 | 0.697 | 0.700 | 0.559 | 0.554 |
| **GBC** | 83.5 | 84.2 | 0.835 | 0.835 | 0.752 | 0.752 |
|  | 81.6 | 82.3 | 0.816 | 0.816 | 0.724 | 0.722 |
| **DT** | 73.8 | 74.2 | 0.738 | 0.736 | 0.607 | 0.607 |
|  | 73.6 | 73.5 | 0.735 | 0.735 | 0.601 | 0.601 |
| **KNN** | 80.7 | 81.0 | 0.807 | 0.805 | 0.710 | 0.710 |
|  | 78.3 | 78.8 | 0.781 | 0.783 | 0.673 | 0.672 |
| **RNC** | 33.8 | 48.1 | 0.341 | 0.296 | 0.007 | 0.007 |
|  | 33.5 | 35.3 | 0.346 | 0.347 | 0.002 | 0.002 |
| **GNB** | 72.0 | 73.3 | 0.720 | 0.715 | 0.584 | 0.579 |
|  | 73.6 | 74.2 | 0.737 | 0.734 | 0.605 | 0.601 |
| **MNB** | 68.5 | 70.1 | 0.685 | 0.684 | 0.530 | 0.527 |
|  | 75.9 | 76.8 | 0.756 | 0.759 | 0.638 | 0.635 |
| **LDA** | 75.2 | 76.5 | 0.752 | 0.750 | 0.631 | 0.628 |
|  | 74.5 | 74.6 | 0.748 | 0.744 | 0.618 | 0.616 |

**Table S7.** Performance of 9 ternary classifiers to predict peptide sequences that can disrupt microbial membranes (MDPs), solely penetrate these membranes (MPPs) to reach intracellular targets or present affinity to larger proteins (PBPs) using 56 physicochemical properties and ADASYN oversampling.

| **Classifier** | **Accuracy** | **Precision** | **Recall** | **F1** | **MCC** | **CK** |
| --- | --- | --- | --- | --- | --- | --- |
| **RFC** | **84.1** | **84.4** | **0.841** | **0.840** | **0.762** | **0.761** |
|  | **83.5** | **83.9** | **0.832** | **0.833** | **0.752** | **0.750** |
| **ETC** | 69.2 | 69.3 | 0.692 | 0.690 | 0.538 | 0.537 |
|  | 69.3 | 69.5 | 0.698 | 0.694 | 0.542 | 0.540 |
| **GBC** | 81.9 | 82.1 | 0.820 | 0.818 | 0.730 | 0.729 |
|  | 79.2 | 79.2 | 0.795 | 0.792 | 0.688 | 0.687 |
| **DT** | 73.6 | 74.1 | 0.736 | 0.737 | 0.604 | 0.604 |
|  | 73.6 | 73.6 | 0.735 | 0.735 | 0.600 | 0.600 |
| **KNN** | 77.7 | 78.2 | 0.777 | 0.776 | 0.666 | 0.665 |
|  | 73.6 | 73.5 | 0.741 | 0.736 | 0.604 | 0.603 |
| **RNC** | 37.7 | 63.0 | 0.375 | 0.273 | 0.083 | 0.062 |
|  | 44.3 | 65.7 | 0.417 | 0.353 | 0.150 | 0.122 |
| **GNB** | 68.6 | 68.4 | 0.688 | 0.672 | 0.537 | 0.530 |
|  | 74.5 | 75.1 | 0.749 | 0.745 | 0.619 | 0.615 |
| **MNB** | 63.5 | 64.9 | 0.635 | 0.632 | 0.456 | 0.452 |
|  | 75.9 | 76.1 | 0.760 | 0.760 | 0.637 | 0.636 |
| **LDA** | 71.9 | 72.9 | 0.720 | 0.713 | 0.584 | 0.579 |
|  | 73.1 | 73.0 | 0.737 | 0.732 | 0.597 | 0.595 |

**Table S8.** Feature importance scores of the 49 physicochemical properties used for the predictive models 1.0-1.5.

| **Features** | **Importance Scores** | | | | |
| --- | --- | --- | --- | --- | --- |
|  | *Model 1.0* | *Model 1.1* | *Model 1.3* | *Model 1.4* | *Model 1.5* |
| *Hydrophobic Ratio* | **0.051** | 0.018 | 0.048 | 0.045 | **0.054** |
| *Cougar* | 0.047 | **0.053** | 0.063 | 0.005 | 0.045 |
| *Molecular weight* | 0.042 | 0.030 | 0.017 | 0.044 | 0.042 |
| *Electrophilicity* | 0.040 | 0.027 | 0.013 | 0.020 | 0.040 |
| *Hydrophobicity Index* | 0.039 | 0.028 | 0.045 | 0.036 | 0.031 |
| *Charge Density* | 0.035 | 0.043 | 0.011 | 0.003 | 0.030 |
| *Aliphatic* | 0.033 | 0.012 | 0.016 | 0.018 | 0.030 |
| *Flexibility* | 0.033 | 0.015 | **0.094** | 0.033 | 0.030 |
| *% Tiny* | 0.030 | 0.011 | 0.025 | **0.077** | 0.033 |
| *ABHPRK* | 0.030 | 0.014 | 0.049 | 0.043 | 0.044 |
| *Amphiphilicity Index* | 0.028 | 0.017 | 0.012 | 0.058 | 0.027 |
| *Instability Index* | 0.027 | 0.034 | 0.016 | 0.010 | 0.030 |
| *H. Ponnuswamy* | 0.025 | 0.014 | 0.055 | 0.030 | 0.030 |
| *H. Welling* | 0.023 | 0.048 | 0.026 | 0.026 | 0.021 |
| *Argos* | 0.023 | 0.010 | 0.018 | 0.011 | 0.022 |
| *Ez* | 0.022 | 0.044 | 0.014 | 0.004 | 0.017 |
| *Acidic* | 0.021 | 0.009 | 0.007 | 0.010 | 0.017 |
| *% Aliphatic* | 0.020 | 0.018 | 0.019 | 0.011 | 0.018 |
| *Isoelectric point* | 0.019 | 0.028 | 0.037 | 0.008 | 0.021 |
| *E. properties 1* | 0.019 | 0.029 | 0.016 | 0.050 | 0.018 |
| *E. properties 2* | 0.019 | 0.063 | 0.013 | 0.029 | 0.025 |
| *H. Cid* | 0.018 | 0.027 | 0.016 | 0.004 | 0.017 |
| *Levitt alpha* | 0.018 | 0.022 | 0.029 | 0.021 | 0.021 |
| *Local flexibility* | 0.017 | 0.019 | 0.018 | 0.004 | 0.015 |
| *H. Wilson* | 0.017 | 0.009 | 0.018 | 0.013 | 0.014 |
| *MSS* | 0.015 | 0.021 | 0.010 | 0.007 | 0.012 |
| *Charge* | 0.015 | 0.018 | 0.020 | 0.007 | 0.015 |
| *PPCALI* | 0.015 | 0.013 | 0.019 | 0.033 | 0.014 |
| *ASHR* | 0.015 | 0.010 | 0.006 | 0.010 | 0.011 |
| *PPPII.coil* | 0.015 | 0.019 | 0.014 | 0.016 | 0.013 |
| *Hmoment* | 0.014 | 0.023 | 0.010 | 0.025 | 0.017 |
| *CCI* | 0.014 | 0.023 | 0.014 | 0.035 | 0.012 |
| *H. Zimmerman* | 0.014 | 0.014 | 0.012 | 0.006 | 0.014 |
| *Bulky properties* | 0.014 | 0.012 | 0.013 | 0.022 | 0.018 |

**Table S8. (suite)**

| **Features** | **Importance Scores** | | | | |
| --- | --- | --- | --- | --- | --- |
|  | *Model 1.0* | *Model 1.1* | *Model 1.3* | *Model 1.4* | *Model 1.5* |
| *Bulkiness* | 0.014 | 0.023 | 0.016 | 0.023 | 0.017 |
| *Aromaticity* | 0.013 | 0.008 | 0.014 | 0.015 | 0.016 |
| *Hardness* | 0.013 | 0.024 | 0.012 | 0.022 | 0.013 |
| *H bonding* | 0.013 | 0.012 | 0.007 | 0.017 | 0.013 |
| *% Small* | 0.013 | 0.010 | 0.014 | 0.021 | 0.014 |
| *AASI* | 0.013 | 0.018 | 0.019 | 0.015 | 0.013 |
| *% Polar* | 0.011 | 0.009 | 0.009 | 0.018 | 0.016 |
| *T scale* | 0.011 | 0.008 | 0.016 | 0.020 | 0.011 |
| *Pepcats* | 0.011 | 0.018 | 0.009 | 0.005 | 0.012 |
| *P in vitro A* | 0.011 | 0.024 | 0.003 | 0.005 | 0.009 |
| *z3* | 0.010 | 0.012 | 0.011 | 0.011 | 0.013 |
| *Refractivity* | 0.009 | 0.014 | 0.013 | 0.005 | 0.011 |
| *H. Jones* | 0.009 | 0.015 | 0.010 | 0.007 | 0.011 |
| *% Acidic* | 0.009 | 0.007 | 0.009 | 0.037 | 0.009 |
| *Penetration depth* | 0.008 | 0.007 | 0.023 | 0.005 | 0.008 |

**Table S9.** Feature importance scores of the 56 physicochemical properties used for the

predictive models 2.0-2.5.

| **Features** | **Importance Scores** | | | | |
| --- | --- | --- | --- | --- | --- |
|  | *Model 2.0* | *Model 2.1* | *Model 2.3* | *Model 2.4* | *Model 2.5* |
| *Molecular Weight* | **0.069** | 0.065 | 0.035 | **0.071** | **0.059** |
| *Charge density* | 0.052 | **0.084** | 0.032 | 0.013 | 0.039 |
| *Acidic* | 0.041 | 0.033 | 0.038 | 0.019 | 0.047 |
| *Cougar* | 0.039 | 0.040 | 0.033 | 0.009 | 0.034 |
| *Electrophilicity* | 0.038 | 0.031 | 0.023 | 0.028 | 0.034 |
| *H bonding* | 0.029 | 0.037 | 0.013 | 0.031 | 0.037 |
| *Aliphatic* | 0.027 | 0.031 | 0.011 | 0.018 | 0.028 |
| *H. Janin* | 0.025 | 0.010 | **0.065** | 0.017 | 0.034 |
| *ASHR* | 0.025 | 0.025 | 0.011 | 0.006 | 0.019 |
| *Local flexibility* | 0.022 | 0.017 | 0.020 | 0.030 | 0.024 |
| *pI* | 0.022 | 0.030 | 0.025 | 0.014 | 0.021 |
| *Aromatic* | 0.022 | 0.030 | 0.005 | 0.019 | 0.015 |
| *% Acidic* | 0.021 | 0.013 | 0.038 | 0.028 | 0.029 |
| *P. in vitro A.* | 0.021 | 0.031 | 0.006 | 0.004 | 0.014 |
| *H. Wolfenden* | 0.021 | 0.020 | 0.019 | 0.005 | 0.016 |
| *Hydrophatic Ratio* | 0.020 | 0.011 | 0.026 | 0.010 | 0.032 |
| *Instability Index* | 0.019 | 0.034 | 0.012 | 0.013 | 0.015 |
| *Flexibility* | 0.017 | 0.011 | 0.039 | 0.023 | 0.026 |
| *% Tiny* | 0.017 | 0.006 | 0.025 | 0.035 | 0.017 |
| *Charge* | 0.017 | 0.019 | 0.018 | 0.010 | 0.018 |
| *MSS* | 0.016 | 0.016 | 0.020 | 0.014 | 0.015 |
| *CCI* | 0.016 | 0.016 | 0.020 | 0.008 | 0.011 |
| *Ez* | 0.016 | 0.014 | 0.011 | 0.012 | 0.015 |
| *Argos* | 0.016 | 0.008 | 0.016 | 0.009 | 0.016 |
| *z3* | 0.016 | .0.012 | 0.016 | 0.027 | 0.013 |
| *Hmoment* | 0.015 | 0.026 | 0.009 | 0.013 | 0.012 |
| *Hardness* | 0.015 | 0.013 | 0.019 | 0.025 | 0.014 |
| *H. Index* | 0.015 | 0.010 | 0.021 | 0.020 | 0.022 |
| *Propensity.to.PPII.coil* | 0.015 | 0.016 | 0.013 | 0.009 | 0.013 |
| *H. Welling* | 0.015 | 0.019 | 0.013 | 0.010 | 0.015 |
| *Amphiphilicity Index* | 0.014 | 0.011 | 0.007 | 0.032 | 0.017 |
| *E. Properties 1* | 0.014 | 0.020 | 0.020 | 0.019 | 0.014 |
| *ABHPRK* | 0.014 | 0.008 | 0.022 | 0.014 | 0.013 |
| *Bulky properties* | 0.013 | 0.013 | 0.023 | 0.016 | 0.011 |
| *Bulkiness* | 0.013 | 0.008 | 0.016 | 0.025 | 0.011 |
| *H. Ponnuswamy* | 0.012 | 0.008 | 0.023 | 0.023 | 0.018 |

**Table S9. (suite)**

| **Features** | **Importance Scores** | | | | |
| --- | --- | --- | --- | --- | --- |
|  | *Model 2.0* | *Model 2.1* | *Model 2.3* | *Model 2.4* | *Model 2.5* |
| *Levitt alpha* | 0.012 | 0.014 | 0.013 | 0.019 | 0.012 |
| *% Aliphatic* | 0.012 | 0.015 | 0.011 | 0.010 | 0.012 |
| *H.Zimmerman* | 0.012 | 0.010 | 0.009 | 0.014 | 0.013 |
| *AASI* | 0.012 | 0.011 | 0.012 | 0.022 | 0.013 |
| *PPCALI* | 0.011 | 0.009 | 0.016 | 0.056 | 0.011 |
| *Refractivity* | 0.011 | 0.012 | 0.010 | 0.013 | 0.008 |
| *Aromaticity* | 0.010 | 0.007 | 0.009 | 0.017 | 0.011 |
| *H. Cid* | 0.010 | 0.007 | 0.019 | 0.010 | 0.011 |
| *E. Properties 2* | 0.010 | 0.020 | 0.015 | 0.028 | 0.011 |
| *T scale* | 0.010 | 0.010 | 0.010 | 0.011 | 0.011 |
| *Penetration depth* | 0.010 | 0.008 | 0.015 | 0.012 | 0.009 |
| *Basic* | 0.009 | 0.016 | 0.008 | 0.015 | 0.011 |
| *% Aromatic* | 0.009 | 0.008 | 0.011 | 0.019 | 0.010 |
| *Pepcats* | 0.009 | 0.008 | 0.008 | 0.006 | 0.009 |
| *% Charged* | 0.009 | 0.009 | 0.011 | 0.008 | 0.010 |
| *H. Jones* | 0.009 | 0.009 | 0.013 | 0.010 | 0.010 |
| *H. Wilson* | 0.009 | 0.006 | 0.020 | 0.014 | 0.011 |
| *% Polar* | 0.009 | 0.007 | 0.012 | 0.013 | 0.012 |
| *% Small* | 0.009 | 0.011 | 0.009 | 0.008 | 0.009 |
| *Linear Moment* | 0.008 | 0.009 | 0.008 | 0.017 | 0.009 |

**Table S10.** Performances of binary and ternary models based on different levels of structure awareness.

| **Binary Models** | | | | | | |
| --- | --- | --- | --- | --- | --- | --- |
| *Subset* | *Model* | *Accuracy (%)* | *Precision* | *Recall* | *Specificity* | *F1* |
| *I* | *1.0* | 73.4 | 0.765 | 0.813 | 0.613 | 0.788 |
| *I* | *1.1* | 81.0 | 0.804 | 0.891 | 0.697 | 0.845 |
| *III* | *1.0* | 57.8 | 0.714 | 0.536 | 0.647 | 0.612 |
| *III* | *1.3* | 51.1 | 0.762 | 0.485 | 0.583 | 0.593 |
| *IV* | *1.0* | 80.0 | n.d. | n.d. | n.d. | n.d. |
| *IV* | *1.4* | 80.0 | n.d. | n.d. | n.d. | n.d. |
| *Complete* | *1.0* | 68.2 | 0.750 | 0.701 | 0.654 | 0.725 |
| *V* | *1.5* | 68.2 | 0.750 | 0.701 | 0.654 | 0.725 |
| **Ternary Models*** | | | | | | |
| *Subset* | *Model* | *Accuracy (%)* | *Precision* | *Recall* | *Specificity* | *F1* |
| *I* | *2.0* | 44.0 | 0.468 | 0.440 | 0.632 | 0.453 |
| *I* | *2.1* | 42.9 | 0.541 | 0.429 | 0.718 | 0.478 |
| *III* | *2.0* | 57.8 | 0.623 | 0.578 | 0.833 | 0.600 |
| *III* | *2.3* | 55.9 | 0.616 | 0.559 | 0.781 | 0.586 |
| *IV* | *2.0* | 65.2 | n.d. | n.d. | n.d. | n.d. |
| *IV* | *2.4* | 80.4 | n.d. | n.d. | n.d. | n.d. |
| *Complete* | *2.0* | 69.5 | 0.696 | 0.695 | 0.827 | 0.695 |
| *V* | *2.5* | 61.8 | 0.653 | 0.618 | 0.821 | 0.635 |

n.d. cannot be determined, *all performance metrics were averaged out.

**References**

1. Osorio, D., Rondón-Villarreal, P. & Torres, R. Peptides: A Package for Data Mining of Antimicrobial Peptides. *R J.* **7**, 4 (2015).

2. Pirtskhalava, M. *et al.* DBAASP v3: database of antimicrobial/cytotoxic activity and structure of peptides as a resource for development of new therapeutics. *Nucleic Acids Res.* **49**, D288–D297 (2021).

3. Müller, A. T., Gabernet, G., Hiss, J. A. & Schneider, G. modlAMP: Python for antimicrobial peptides. *Bioinformatics* **33**, 2753–2755 (2017).

4. Eisenberg, D., Weiss, R. M. & Terwilliger, T. C. The hydrophobic moment detects periodicity in protein hydrophobicity. *Proc. Natl. Acad. Sci.* **81**, 140–144 (1984).

5. Liang, G. & Li, Z. Factor Analysis Scale of Generalized Amino Acid Information as the Source of a New Set of Descriptors for Elucidating the Structure and Activity Relationships of Cationic Antimicrobial Peptides. *QSAR Comb. Sci.* **26**, 754–763 (2007).

6. Cruciani, G. *et al.* Peptide studies by means of principal properties of amino acids derived from MIF descriptors. *J. Chemom.* **18**, 146–155 (2004).

7. Juretić, D., Vukičević, D., Ilić, N., Antcheva, N. & Tossi, A. Computational Design of Highly Selective Antimicrobial Peptides. *J. Chem. Inf. Model.* **49**, 2873–2882 (2009).

8. Collantes, E. R. & Dunn, W. J. Amino Acid Side Chain Descriptors for Quantitative Structure-Activity Relationship Studies of Peptide Analogs. *J. Med. Chem.* **38**, 2705–2713 (1995).

9. Raychaudhury, C., Banerjee, A., Bag, P. & Roy, S. Topological Shape and Size of Peptides: Identification of Potential Allele Specific Helper T Cell Antigenic Sites. *J. Chem. Inf. Comput. Sci.* **39**, 248–254 (1999).

10. Zaliani, A. & Gancia, E. MS-WHIM Scores for Amino Acids: A New 3D-Description for Peptide QSAR and QSPR Studies. *J. Chem. Inf. Comput. Sci.* **39**, 525–533 (1999).

11. Koch, C. P. *et al.* Scrutinizing MHC-I Binding Peptides and Their Limits of Variation. *PLoS Comput. Biol.* **9**, e1003088 (2013).

12. Cocchi, M. & Johansson, E. Amino Acids Characterization by GRID and Multivariate Data Analysis. *Quant. Struct.-Act. Relatsh.* **12**, 1–8 (1993).

13. Zhao, G. & London, E. An amino acid “transmembrane tendency” scale that approaches the theoretical limit to accuracy for prediction of transmembrane helices: Relationship to biological hydrophobicity. *Protein Sci.* **15**, 1987–2001 (2006).

14. Hellberg, S., Sjoestroem, M., Skagerberg, B. & Wold, S. Peptide quantitative structure-activity relationships, a multivariate approach. *J. Med. Chem.* **30**, 1126–1135 (1987).

15. Sandberg, M., Eriksson, L., Jonsson, J., Sjöström, M. & Wold, S. New Chemical Descriptors Relevant for the Design of Biologically Active Peptides. A Multivariate Characterization of 87 Amino Acids. *J. Med. Chem.* **41**, 2481–2491 (1998).
